# Perioperative Anxiety in Total Knee Arthroplasty: Clinical Consequences, Potential Mechanisms, and Management—A Narrative Review

**DOI:** 10.3390/jcm15208140

**Published:** 2026-10-20

**Authors:** Honghua Zhang, Xiang Ji, Xiangxiang Sun, Zichao He, Haotian Yi, Yanling Yang, Huanli Bao, Yuanchi Huang, Jianbing Ma

**Affiliations:** 1 Department of Joint Surgery, Honghui Hospital, Xi’an Jiaotong University, Xi’an 710054, China; 15229984590@163.com (H.Z.);; 2 Yan’an Medical College, Yan’an University, Yan’an 716000, China

**Keywords:** total knee arthroplasty, anxiety, perioperative period, pain, function

## Abstract

Total knee arthroplasty (TKA) treats end-stage knee osteoarthritis, yet some patients report persistent pain, incomplete recovery, or dissatisfaction. Perioperative anxiety is a potentially modifiable factor associated with poorer outcomes. This narrative review examines its clinical consequences, mechanisms, and management in TKA. PubMed, Embase, Web of Science, and the China National Knowledge Infrastructure were searched without date or language restrictions through 25 September 2026. Evidence was stratified by anxiety-related selection at enrollment: studies of anxiety-selected patients provided direct evidence, whereas studies without such selection provided indirect evidence, regardless of whether anxiety was a primary, secondary, or exploratory outcome. Sixty studies underwent formal risk-of-bias appraisal. Higher anxiety was associated with greater postoperative pain and analgesic requirements and poorer patient-reported recovery and satisfaction, whereas findings for persistent pain and long-term function were less consistent. Proposed pathways include catastrophizing, kinesiophobia, reduced self-efficacy, pain sensitization, and stress-related biological processes. Pharmacological, psychoeducational, cognitive behavioral, digital, and adjunctive interventions may reduce anxiety, but direct evidence remains limited, and improvements do not consistently translate into less pain or opioid use, better function, or higher satisfaction. Anxiety may be considered in preoperative risk assessment, while standardized assessment and higher-quality trials remain needed.

## 1. Introduction

Knee osteoarthritis (KOA) is a common degenerative joint disorder that causes knee pain, deformity, and restricted mobility, substantially impairing patients’ quality of life [1]. The prevalence of KOA continues to increase, placing a considerable burden on families and society [2]. For patients with end-stage KOA who have severe symptoms and derive inadequate benefit from conservative treatment, total knee arthroplasty (TKA) can effectively relieve pain and improve joint function [3]. However, some patients remain dissatisfied with the outcome of surgery, mainly because of inadequate postoperative pain relief or suboptimal functional recovery [4].

Despite continuing advances in surgical techniques, implant design, and perioperative management, interindividual differences in postoperative pain, function, and satisfaction cannot be fully explained by technical factors alone [5]. Perioperative anxiety is a relatively common and potentially modifiable psychological factor [6,7]. Previous studies have found that higher anxiety levels are associated with greater postoperative pain, less functional improvement, lower patient satisfaction, and poorer outcomes on some hospitalization-related measures [8,9,10]. However, definitions of anxiety, assessment instruments, adjustment for confounding factors, and the timing of outcome measurement vary across studies, limiting direct comparison of their findings. Evidence from anxiety-screened patients is often considered together with evidence from the general TKA populations. However, anxiety was not used to select participants in many of these general-population studies. These two types of evidence address different clinical questions and should not be interpreted as equivalent.

Accordingly, this narrative review examines the associations between perioperative anxiety and clinical outcomes after TKA. It also summarizes proposed cognitive, behavioral, and biological mechanisms and evaluates current management strategies with attention to the directness of the available evidence. We distinguish evidence from anxiety-screened populations from that in unselected TKA populations. This distinction helps clarify the clinical relevance and limitations of the current evidence and identify priorities for future research.

## 2. Literature Search and Evidence Classification

The literature search was conducted in PubMed, Web of Science Core Collection, Embase, and the China National Knowledge Infrastructure (CNKI) between 5 May and 25 September 2026. The searches combined TKA- and anxiety-related terms using database-specific fields and Boolean operators, as detailed in Supplementary Table S1. No publication-date or language restrictions were applied. In Embase, only records categorized as Articles (*n* = 1268) were considered for screening. Studies were considered relevant if they addressed perioperative anxiety or anxiety-related psychological risk in adults undergoing TKA and reported relevant clinical outcomes or evaluated a perioperative intervention. Mixed total hip arthroplasty (THA)/TKA studies were retained when relevant, with TKA-specific findings considered separately when available. Studies examining related psychological or pain-processing factors, including pain catastrophizing, kinesiophobia, self-efficacy, and pain sensitization, were also considered when relevant to the discussion of potential mechanisms. Studies that did not directly assess anxiety were used to provide mechanistic context rather than evidence of anxiety-specific effects. Titles and abstracts were screened for relevance, followed by full-text review of potentially eligible reports (Table S1). The database searches yielded 3141 records, of which 2035 remained after duplicate removal. Following relevance screening, 262 records were retained for further consideration. Ultimately, 82 references were included in this narrative review, including 60 studies that underwent formal risk-of-bias appraisal.

Evidence was synthesized using a stratified narrative approach based on anxiety-related patient selection at enrollment. Direct evidence came from intervention studies enrolling patients with anxiety identified through screening, a pre-existing anxiety diagnosis, or predefined elevated psychological risk explicitly including anxiety. Studies involving combined anxiety and depression or broader psychological risk were distinguished from anxiety-only populations. Indirect evidence came from general TKA or mixed arthroplasty populations without anxiety-based selection. This classification was independent of whether anxiety was assessed as a primary, secondary, or exploratory outcome. Systematic reviews, meta-analyses, and measurement studies informed the background, while study protocols were used to describe ongoing research rather than establish efficacy. For the main synthesis, information on study design, population, anxiety assessment, intervention or exposure, clinical outcomes, follow-up, and principal findings was extracted. Conflicting findings were interpreted in light of population directness, study design, risk of bias, anxiety measures, outcome definitions, and follow-up duration rather than being quantitatively pooled across heterogeneous studies.

Risk of bias was independently assessed by two reviewers using design-appropriate appraisal tools. Randomized trials were evaluated using the revised Cochrane risk-of-bias tool for randomized trials (RoB 2), including the cluster-randomized extension where applicable; non-randomized intervention studies were assessed using Risk Of Bias In Non-randomized Studies of Interventions (ROBINS-I); prognostic-factor studies were assessed using Quality In Prognosis Studies (QUIPS); and the relevant Joanna Briggs Institute (JBI) critical appraisal checklists were used for other observational or quasi-experimental studies. Formal risk-of-bias appraisal was limited to original studies contributing empirical findings on perioperative anxiety, associated clinical outcomes, interventions, or related mechanisms, for which a design-appropriate appraisal tool could be applied. Of the 82 cited references, 60 studies underwent formal appraisal: 23 randomized trials, four non-randomized intervention studies, 23 prognostic-factor studies, and 10 other observational or quasi-experimental studies. Systematic reviews, meta-analyses, study protocols, and references used solely for background or measurement information were not formally appraised. Fuchs et al. was retained for descriptive discussion but excluded from formal appraisal because its allocation method could not be reliably classified [11]. RoB 2 and ROBINS-I judgments were outcome- and comparison-specific, whereas QUIPS results were interpreted at the domain level and JBI results at the checklist-item level. Because these tools use different appraisal frameworks, their results were interpreted separately rather than converted into a common quality score. Detailed assessments are provided in Supplementary Table S2. Grey literature and unpublished studies were not systematically searched; publication bias therefore cannot be excluded.

## 3. Definition and Assessment of Perioperative Anxiety

Anxiety related to TKA may manifest as a transient increase in state anxiety within the perioperative context or reflect relatively stable trait anxiety or a pre-existing anxiety disorder. An elevated anxiety-scale score does not, by itself, constitute a clinical diagnosis of an anxiety disorder [12,13]. Anxiety also frequently coexists with depression, pain catastrophizing, kinesiophobia, and sleep disturbance [6,14]. Therefore, observed adverse outcomes cannot simply be attributed to anxiety alone.

Anxiety at different stages of the perioperative period may have different triggers and clinical implications. Interpretation of study findings should therefore consider the type of anxiety, the assessment instrument, and the timing of measurement. Instruments commonly used in TKA research include the Hospital Anxiety and Depression Scale–Anxiety subscale (HADS-A), Generalized Anxiety Disorder-7 (GAD-7), State–Trait Anxiety Inventory (STAI), Hamilton Anxiety Rating Scale (HAMA), and Amsterdam Preoperative Anxiety and Information Scale (APAIS). These instruments assess different aspects of anxiety, and their reported cutoff values may be used for screening or symptom classification rather than for establishing a psychiatric diagnosis. The HADS-A is commonly used to screen anxiety symptoms in patients with physical illness, whereas the GAD-7 provides a brief assessment of anxiety symptom severity. The STAI distinguishes state from trait anxiety, the HAMA is clinician-rated and assesses anxiety severity, and the APAIS focuses on surgery- and anesthesia-related anxiety and patients’ information needs [12,15,16,17,18]. Differences in the constructs assessed, cutoff values, and assessment time points are important reasons why findings across studies are difficult to compare directly (Table 1).

## 4. Perioperative Anxiety and Outcomes After TKA

### 4.1. Anxiety and Pain

Existing studies generally suggest that higher preoperative anxiety is associated with more severe acute postoperative pain after TKA, whereas evidence for persistent pain is less consistent [7,9,23]. In a longitudinal cohort including patients undergoing TKA or THA, Paredes et al. reported that preoperative anxiety predicted acute pain 48 h after TKA [24]. Croppi et al. found that preoperative trait anxiety and pain catastrophizing were associated with chronic pain 6 months after TKA [25]. Greater analgesic requirements have also been reported in patients with higher anxiety during hospitalization or the early postoperative period [26]. In a retrospective study of 4404 patients undergoing primary TKA, Rivas et al. found that patients with a pre-existing anxiety diagnosis reported greater pain at rest and during activity and had higher inpatient opioid consumption [27]. Anxiety may therefore be one component of a broader psychological and clinical risk profile for postoperative pain. Consistent with this interpretation, a systematic review of 37 TKA studies found that associations between psychological factors, including anxiety, depression, and pain catastrophizing, and chronic pain were not consistent across studies [28].

Associations with opioid use and persistent pain also appear to depend on when anxiety is measured and which covariates are considered. Larach et al. found that preoperative anxiety was associated with opioid use at 6 weeks after adjustment for concurrent pain, but the association was no longer significant after adjustment for preoperative opioid use [29]. Terradas-Monllor et al. reported that anxiety measured 1 week after knee arthroplasty was associated with chronic postsurgical pain in univariable analysis but was not retained in the final multivariable models [30]. These findings do not establish a consistent independent effect of anxiety on persistent pain.

### 4.2. Anxiety and Functional Recovery

Beyond pain outcomes, anxiety is associated with some aspects of postoperative functional recovery. Patient-reported or subjectively perceived recovery includes patients’ own ratings of function, confidence in movement, fear of mobilization, and perceived ability to participate in rehabilitation. Varış et al. found that higher preoperative anxiety was associated with less improvement in some functional outcomes and poorer subjective assessments of recovery [26]. Patients with anxiety may also experience greater pain, kinesiophobia, or concerns about early mobilization and exercise, which may reduce participation in rehabilitation [31,32,33]. Cross-sectional studies have linked anxiety with self-efficacy, while pain catastrophizing and kinesiophobia have been associated with exercise adherence [34,35]. These findings involve related but distinct psychological constructs and do not establish that anxiety independently delays physical recovery.

Objective functional recovery should be considered separately. It is typically assessed using performance-based or physical measures such as muscle strength, range of motion, walking performance, and timed mobility tests. Hiyama et al. reported that discharge anxiety was associated with poorer functional outcomes at 6 months [36], whereas Jiménez Ortiz et al. found no differences in 1 year Knee Society Score functional improvement across preoperative anxiety groups [37]. More directly, Aalders et al. found no differences at 12 months in knee extension strength, range of motion, or timed up-and-go performance between patients with and without preoperative anxiety symptoms after adjustment [38]. These findings suggest that poorer subjective recovery or greater psychological distress should not automatically be interpreted as persistent impairment in objective physical function.

### 4.3. Anxiety, Satisfaction, Quality of Life, and Healthcare Utilization

Patient satisfaction, quality of life, and other patient-reported outcomes reflect subjective aspects of recovery after TKA. Patients with anxiety symptoms or broader psychological distress have been reported to experience lower satisfaction and poorer patient-reported outcomes [39,40]. Jia et al. similarly found poorer patient-reported outcomes, greater dissatisfaction, and higher healthcare utilization in patients with a pre-existing psychiatric diagnosis [41], while adverse subjective outcomes have also been reported at mid- to long-term follow-up [42,43]. Evidence for healthcare utilization and complications is less consistent. Rivas et al. found that a higher unadjusted rate of discharge to rehabilitation or skilled nursing facilities among patients with anxiety was no longer significant after adjustment [27]. Zalikha et al. reported associations of combined anxiety and depression with perioperative complications and adverse outcomes [10]. Because several studies assessed anxiety together with depression or broader psychiatric conditions, these findings should not be interpreted as anxiety-specific effects. Anxiety symptoms may also change after surgery. Mahdi et al. reported that 59% of patients with preoperative anxiety symptoms no longer met the HADS-A symptom threshold 1 year after TKA [44]. Although the uncontrolled design limits causal interpretation, the finding suggests that preoperative anxiety symptoms do not necessarily persist after surgery.

The evidence reviewed above indicates that perioperative anxiety often coexists with depression, pain catastrophizing, kinesiophobia, sleep disturbance, and pain sensitization. The consistency of evidence varies across outcomes. Overall, higher anxiety levels are associated with greater postoperative pain, poorer patient-reported recovery, and lower satisfaction, whereas associations with objective function and healthcare utilization are less consistent. Anxiety assessment may therefore be considered as one component of comprehensive preoperative risk assessment and may help identify patients who require further evaluation or perioperative support.

## 5. Potential Mechanisms Linking Anxiety to Outcomes After TKA

### 5.1. Pain-Related Cognitions, Expectations, and Catastrophizing

Pain-related cognitions may represent one pathway linking anxiety with postoperative pain after TKA. Higher anxiety has been associated with negative pain expectations, hypervigilance, and pain catastrophizing [45,46,47]. Preoperative pain catastrophizing is also associated with more severe postoperative pain, and anxiety and catastrophizing often coexist in unfavorable pain trajectories [48,49]. However, these constructs should not be assumed to form a single causal pathway. Paredes et al. found no significant mediating effect of pain catastrophizing on the association between preoperative anxiety and acute postoperative pain after TKA [24]. Negative expectations and catastrophizing are therefore better regarded as related cognitive factors and potential intervention targets rather than established mediators. Interventions targeting these processes may include strategies to reduce catastrophic thinking, modify negative pain expectations, and decrease excessive attention to pain [6,50].

### 5.2. Kinesiophobia, Self-Efficacy, and Rehabilitation Behaviors

Anxiety may interact with kinesiophobia, activity avoidance, and low confidence in rehabilitation, potentially affecting participation in early mobilization and rehabilitation exercises [51]. When anxiety coexists with higher pain levels, patients may be more reluctant to perform out-of-bed mobilization, active range-of-motion exercises, or weight-bearing activities [32,52,53]. Anxiety has also been associated with self-efficacy, while pain catastrophizing and kinesiophobia have been linked to exercise adherence [34,35]. Lower self-efficacy is associated with poorer recovery outcomes [54]. However, these constructs are distinct, and most available evidence is cross-sectional or observational. A fixed causal pathway linking anxiety, self-efficacy, kinesiophobia, and rehabilitation behavior therefore cannot be established.

### 5.3. Pain Sensitization and Persistent Postsurgical Pain

Beyond cognitive and behavioral factors, anxiety may coexist with abnormal pain processing and central sensitization and may be associated with persistent postsurgical pain (PPSP) [55,56]. In patients with psychological distress undergoing TKA, concomitant central sensitization has been associated with poorer long-term function and greater opioid use [23]. Rice et al. reported that preoperative psychological disorders were associated with chronic pain after TKA, with anxiety disorders specifically associated with moderate-to-severe pain at 6–12 months [57]. Anxiety has also been associated with pain trajectories extending from the acute to persistent phase [48]. However, current evidence does not establish that anxiety causes central sensitization or that central sensitization mediates the relationship between anxiety and TKA outcomes. Central sensitization is therefore better regarded as a candidate mechanism and potential risk marker rather than an established mediator.

### 5.4. Stress-Related Biological Processes

Stress-related biological processes may represent another potential pathway linking preoperative anxiety with postoperative pain. In a longitudinal study of 91 patients undergoing unilateral TKA, Bruehl et al. found that perioperative oxidative stress was associated with preoperative psychological characteristics and pain outcomes at 6 months [58]. Oxidative stress, indexed by F2-isoprostanes and isofurans, partially mediated the association between preoperative anxiety and postoperative pain, whereas indices of central sensitization showed associations that were independent of oxidative stress. These findings suggest that anxiety, pain sensitization, and oxidative stress may reflect partially overlapping pathways. However, the small sample and secondary mechanistic analysis limit causal interpretation, and oxidative stress should therefore be regarded as a candidate mediator requiring further validation.

**Figure 1:**
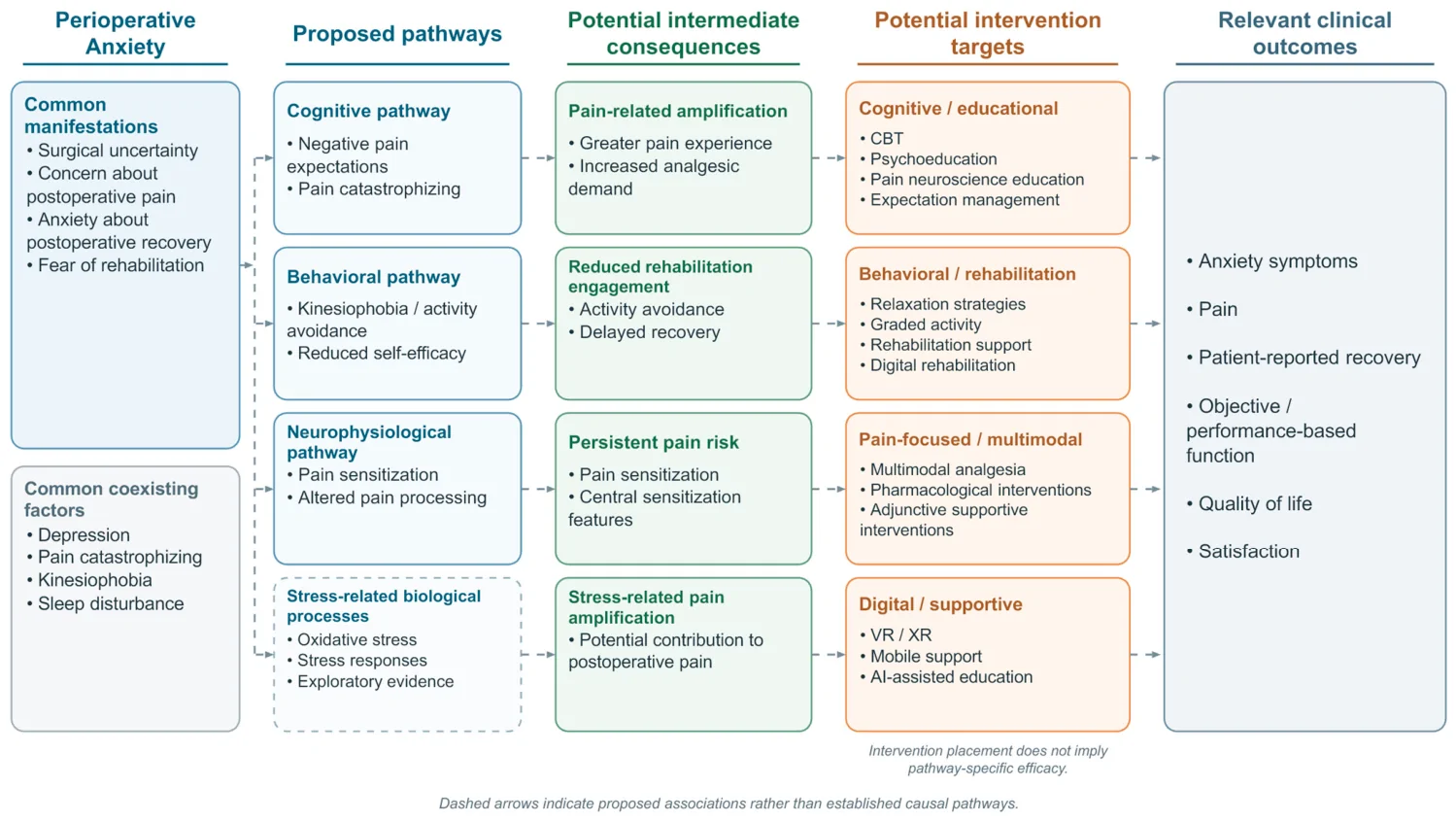
Potential mechanisms and intervention targets linking perioperative anxiety to outcomes after total knee arthroplasty. Perioperative anxiety may be associated with postoperative pain and recovery through interacting cognitive, behavioral, neurophysiological, and stress-related biological processes. Stress-related biological processes are supported mainly by exploratory evidence. Dashed arrows indicate proposed associations rather than established causal pathways, and intervention placement does not imply pathway-specific efficacy. Anxiety, depression, pain catastrophizing, kinesiophobia, and self-efficacy represent distinct constructs. Patient-reported recovery and objective or performance-based function should be considered separately. CBT, cognitive behavioral therapy; TKA, total knee arthroplasty; VR, virtual reality; XR, extended reality.

Current evidence suggests that anxiety may be associated with TKA outcomes through interrelated cognitive, behavioral, and neurophysiological processes. Negative expectations and catastrophizing may amplify pain, while kinesiophobia, activity avoidance, and low self-efficacy may reduce participation in rehabilitation. Pain sensitization may also be associated with persistent pain. However, these proposed pathways are derived mainly from observational studies and statistical mediation analyses and do not establish a unified causal model. Intervention studies should therefore assess not only anxiety symptoms but also relevant process measures, such as pain-related cognitions, activity engagement, and pain sensitization, to determine whether changes in these factors accompany changes in clinical outcomes (Figure 1).

## 6. Evidence for the Management of Perioperative Anxiety in Patients Undergoing TKA

### 6.1. Pharmacological Interventions

Pharmacological evidence can be divided into direct and indirect evidence according to whether anxiety was used to select participants. Direct evidence comes from studies that enrolled patients with anxiety or elevated psychological risk and evaluated targeted pharmacological interventions. Indirect evidence comes mainly from analgesic or multimodal drug studies enrolling general TKA patients without anxiety-based selection. Anxiety was commonly assessed as a secondary outcome in these studies. Direct evidence is more relevant to patients with identified anxiety but is limited by small samples, whereas indirect evidence cannot establish treatment efficacy specifically in patients with anxiety.

Among studies classified as direct evidence, Yılmaz et al. enrolled TKA patients with mild-to-moderate anxiety or depressive symptoms. Alprazolam combined with standard analgesia was associated with lower postoperative analgesic requirements and better knee function at 1 month [59]. Liang et al. similarly reported improvements in anxiety and pain, reduced analgesic use, and higher satisfaction after alprazolam, but no additional improvement in joint function [60]. These studies therefore provide direct intervention evidence, although their mixed anxiety/depression populations limit anxiety-specific interpretation. Jiang et al. evaluated short-term, low-dose olanzapine in patients undergoing primary THA or TKA with preoperative STAI-S scores ≥ 40. The intervention was associated with lower perioperative anxiety, improved sleep, less early postoperative pain, and lower opioid consumption [61]. However, functional benefits were mainly observed in the THA subgroup, limiting direct extrapolation to TKA patients with anxiety.

Indirect evidence comes mainly from analgesic studies in general TKA populations. Zheng et al. reported lower HADS scores, less pain, better early function, and a shorter hospital stay with multimodal analgesia [62]. Sarridou et al. similarly found lower perioperative pain and STAI scores with parecoxib [63]. However, neither study selected participants according to anxiety status, so these findings cannot establish efficacy specifically in patients with anxiety. In contrast, YaDeau et al. found that duloxetine reduced opioid consumption but did not improve ambulatory pain or anxiety scores on postoperative day 14 [64]. These findings suggest that pain, analgesic use, and anxiety may change in parallel in some studies, but analgesic benefit does not consistently translate into reduced anxiety.

The reported benefits require consideration of study-specific bias. Yılmaz et al. [59] had a serious risk of bias due to nonrandom allocation and inadequate confounding control, while the early anxiety improvement reported by Zheng et al. [62] was at high risk of bias because of substantial postrandomization exclusions. YaDeau et al. [64] found reduced opioid use without an anxiety benefit, although the prespecified analysis of this secondary outcome could not be verified (RoB 2: some concerns). Overall, some pharmacological interventions may reduce short-term anxiety or analgesic requirements, but direct evidence remains limited by small studies and mixed THA/TKA populations. Sustained benefits for pain, function, and satisfaction remain uncertain. Current evidence does not support routine anxiolytic use, and treatment should be individualized according to anxiety severity, psychiatric history, comorbidities, and medication-related risks.

### 6.2. Psychoeducational and Cognitive Behavioral Interventions

Psychoeducational and cognitive behavioral interventions aim to reduce perioperative uncertainty, modify maladaptive cognitions, and improve coping with pain and rehabilitation demands. These approaches avoid medication-related risks and may be relatively easy to implement. However, their effects on mid- to long-term anxiety and clinical recovery remain uncertain.

#### 6.2.1. Psychoeducation

Preoperative psychoeducation provides information about the surgical process, pain management, and rehabilitation goals and may reduce uncertainty about what to expect. Direct evidence in patients identified through anxiety screening remains limited, and most available studies therefore provide indirect evidence. Medina-Garzón et al. reported that three nurse-led preoperative motivational interviewing sessions reduced preoperative anxiety, although postoperative pain and functional outcomes were not assessed [65]. In a systematic review and meta-analysis of 19 randomized controlled trials, Lloyd et al. found a small reduction in anxiety and improvements in patient knowledge and postoperative pain, but no clear effects on time to discharge, quality of life, or complications [66]. Education studies have also addressed related psychological outcomes: nurse-led education has been evaluated for kinesiophobia, whereas pain neuroscience education did not provide additional improvement in pain or anxiety [53,67]. Because most studies involved general TKA populations and used heterogeneous educational content and delivery, psychoeducation is best regarded as a supportive strategy; its long-term effects in patients with identified anxiety remain uncertain.

#### 6.2.2. Cognitive Behavioral Therapy (CBT)

Cognitive behavioral therapy (CBT) aims to improve coping with surgery, pain, and rehabilitation by modifying maladaptive cognitions. Direct evidence remains limited. In an exploratory trial of TKA patients selected for elevated psychological symptoms, Kaynar et al. found lower preoperative emotional symptom scores after 1 month of RxWell digital CBT, but the final analytic sample was very small and postoperative clinical outcomes were not assessed [19]. The subsequent SuRxgWell study remains at the protocol stage and therefore provides no efficacy evidence [68].

Evidence from the general populations also shows that anxiety reduction does not necessarily translate into better clinical recovery. Chen et al. reported lower anxiety scores with CBT on postoperative days 7 and 14 in patients aged ≥ 70 years, without additional improvements in pain or knee function [20]. In a pilot study, Pester et al. found lower pain severity and interference at 6 weeks after mindfulness-based CBT, but the differences were not sustained at 3 or 6 months; mediation analyses implicated changes in pain catastrophizing rather than anxiety [69]. Hanley et al. also reported short-term reductions in anxiety and pain after mindfulness or hypnotic suggestion in a mixed THA/TKA population, although these brief interventions differed from standardized CBT [50]. Overall, evidence for CBT specifically in anxiety-screened TKA patients remains limited, and short-term improvements in anxiety have not consistently translated into sustained pain or functional benefits.

The evidence is further limited by study-specific bias. Kaynar et al. [19] and Chen et al. [20] had high-risk judgments for anxiety outcomes, mainly due to postrandomization exclusions and missing data, respectively. The short-term pain benefit reported by Pester et al. [69] was subject to serious risk of bias because of historical controls and residual confounding. Although psychoeducational and cognitive behavioral interventions appear feasible in perioperative TKA care, most studies did not specifically enroll patients with anxiety, and the clinical relevance and durability of their effects remain uncertain. Differences in intervention delivery and adherence also complicate interpretation. Future trials should focus on anxiety-selected patients and assess whether reductions in anxiety are accompanied by meaningful improvements in pain, rehabilitation participation, and long-term function.

### 6.3. Digital Interventions

Digital interventions include virtual reality (VR) and extended reality (XR), mobile app-based telerehabilitation, and artificial intelligence (AI)-assisted patient education. Direct evidence is largely limited to small proof-of-concept studies of digital CBT in patients selected for elevated psychological symptoms, whereas most mobile rehabilitation and VR/XR studies enrolled general TKA populations without anxiety-based selection. Digital technologies may extend perioperative support through education, symptom monitoring, rehabilitation guidance, and feedback across the preoperative and postoperative periods. However, the digital platform itself should not be regarded as an effective psychological intervention; clinical effects depend on the intervention content, mode of delivery, and patient engagement.

Evidence for VR/XR and mobile digital interventions is mixed. Fuchs et al. reported reductions in anxiety after early VR-assisted physiotherapy, but these improvements did not differ significantly from conventional physiotherapy, and no additional functional benefit was observed at 6 months. Although described as randomized, the study reported that patients were “time randomly assigned” without clarifying the allocation mechanism. This uncertainty prevented reliable classification for formal risk-of-bias appraisal, so the findings were retained for descriptive discussion but not considered evidence of VR superiority [11]. A systematic review and meta-analysis similarly suggested small short-term improvements in pain and anxiety with XR, although substantial heterogeneity and inconsistent effects on objective function and quality of life limit interpretation [70]. In 108 patients undergoing primary unilateral TKA, Kara and Karabulut found that a multicomponent mobile intervention improved anxiety, pain, kinesiophobia, sleep, and early function; however, only the between-group difference in kinesiophobia reached the minimal clinically important difference (MCID) threshold [71]. Gan et al. reported lower perioperative anxiety and greater satisfaction with physician-supervised, ChatGPT 4.0-assisted informed consent, but no corresponding improvement in pain or function [21]. These studies therefore suggest short-term psychological benefits, but their clinical significance and durability remain uncertain.

Study-specific bias limits the interpretation of these findings. In Gan et al. [21], the immediate postconsent anxiety outcome had some concerns, while the POD5 result was at high risk of bias because of missing data. Kara and Karabulut [71] also had some concerns for week-4 anxiety, and the between-group difference did not reach the study-defined MCID. These limitations weaken the evidence for clinically meaningful or sustained benefits. Digital tools may extend perioperative education, psychological support, and rehabilitation guidance, but their superiority over conventional care remains unproven. Evaluation should focus on intervention content, adherence, clinical oversight, information quality, and relevant clinical outcomes rather than usage or patient satisfaction alone.

### 6.4. Other Adjunctive Interventions

Beyond pharmacological, psychological and behavioral, and digital interventions, adjunctive approaches such as music therapy, acupuncture, auricular acupoint stimulation, aromatherapy, and nursing support have also been evaluated in patients undergoing TKA. These approaches generally aim to promote relaxation, redirect attention, or improve the perioperative pain experience, and many are relatively easy to implement. Reported benefits are largely limited to immediate preoperative or short-term in-hospital outcomes. Interpretation is further limited by heterogeneous intervention protocols, difficulties with blinding, and possible placebo or provider-related effects.

Direct evidence for adjunctive interventions remains limited. In anxiety-screened patients undergoing hip or knee arthroplasty, Kainkaryam et al. reported an immediate reduction in anxiety after a single preoperative acupuncture session, but post-intervention anxiety was not assessed in the control group and postoperative pain, opioid use, and clinical recovery did not differ between groups [72]. In a small TKA pilot trial, Tailor et al. found no significant benefit of lavender–peppermint aromatherapy for postoperative anxiety or pain, despite favorable satisfaction and adherence [22].

Most other evidence is indirect and focuses on short-term outcomes. Auricular acupressure was associated with lower perioperative anxiety, better sleep, and less short-term pain in a mixed surgical observational cohort, but did not clearly improve hospital stay or complications [73]. Music-based interventions have generally shown short-term reductions in anxiety and pain [74,75]; in the trial by Nalbant et al., improvements in postoperative STAI-S and quality of recovery exceeded the reported minimal clinically important difference thresholds [75]. Other small trials reported lower early anxiety after diaphragmatic breathing [76] or noise-control/music interventions [77], whereas guided imagery did not improve state anxiety [78]. Emotional freedom technique reduced immediate postoperative anxiety and pain, but substantial post-randomization loss and unblinded assessment limit interpretation [79]. Overall, these findings are mainly short term and cannot be directly extrapolated to patients with persistent or clinically diagnosed anxiety.

The appraisal findings warrant caution when interpreting these results. Kainkaryam et al. [72] did not assess postintervention anxiety in the control group, so the observed reduction cannot establish a comparative benefit. Although Nalbant et al. [75] reported anxiety improvements exceeding the stated MCID thresholds, postrandomization exclusions led to a high-risk judgment. Tanriver et al. [79] also had a high-risk judgment due to substantial exclusions and unblinded outcome assessment. Although adjunctive interventions are generally easy to implement and carry a low treatment burden, evidence of benefit is mainly limited to short-term outcomes. Future trials should use appropriate controls, prespecified primary outcomes, and assess clinical relevance alongside statistical significance.

### 6.5. Summary of the Intervention Evidence

**Figure 2:**
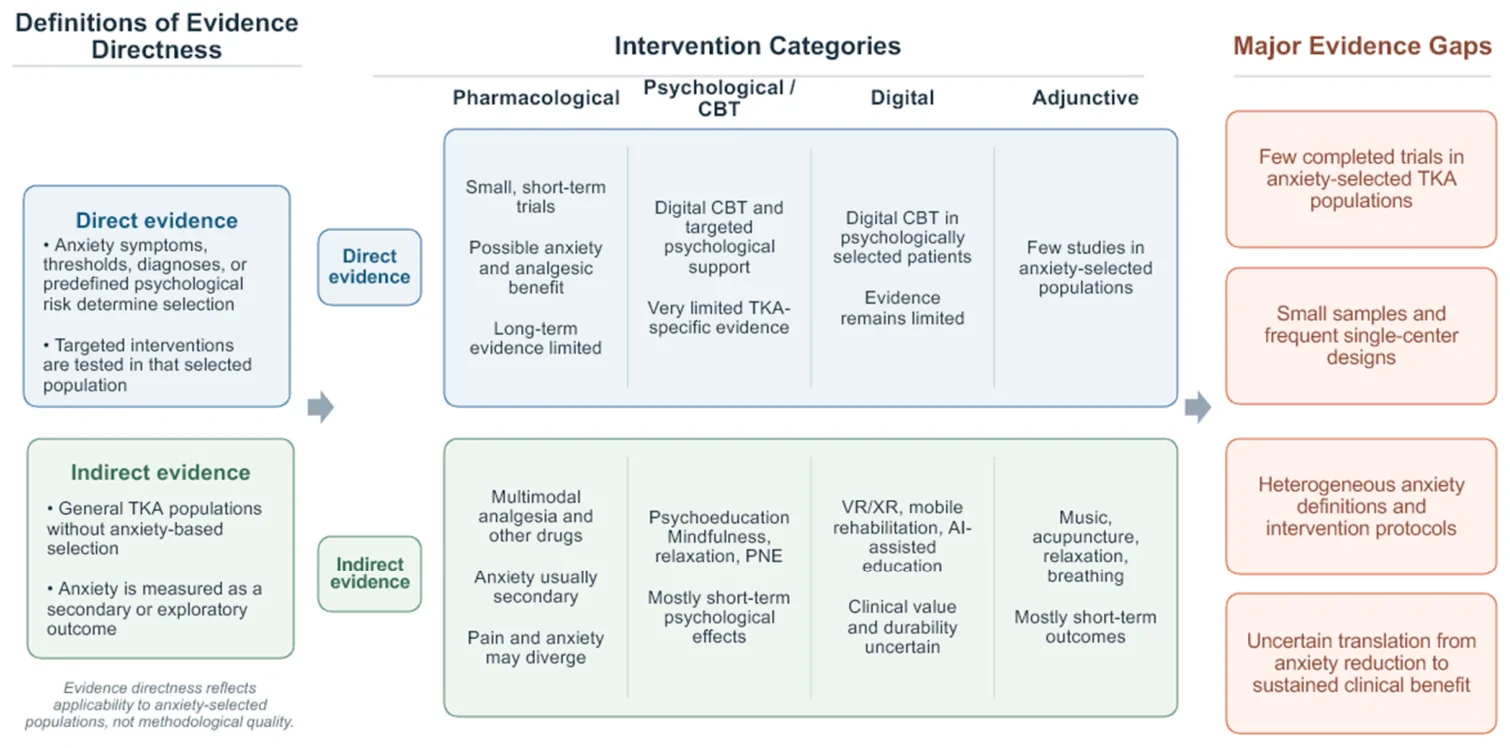
Evidence stratification of studies evaluating perioperative anxiety interventions in total knee arthroplasty. Direct evidence refers to studies enrolling patients selected according to anxiety symptoms, an anxiety-scale threshold, an anxiety diagnosis, or predefined psychological risk explicitly including anxiety. Indirect evidence derives from general TKA or mixed arthroplasty populations without anxiety-based selection. Evidence classification was determined by patient selection at enrollment, regardless of whether anxiety was assessed as a primary, secondary, or exploratory outcome. References to secondary or exploratory anxiety outcomes in the figure describe common study characteristics rather than classification criteria. Anxiety-only, anxiety–depression, and broader psychological-risk populations were not considered equivalent. Evidence directness reflects applicability to anxiety-selected populations rather than methodological quality. The figure summarizes major intervention categories and limitations of the current evidence. AI, artificial intelligence; CBT, cognitive behavioral therapy; PNE, pain neuroscience education; TKA, total knee arthroplasty; VR, virtual reality; XR, extended reality.

Risk-of-bias assessments informed the narrative synthesis, with greater emphasis placed on interpretable between-group comparisons with fewer methodological limitations. Findings affected by serious confounding, substantial missing data, or uncontrolled within-group changes were interpreted more cautiously. Differences in study populations, outcomes, and follow-up were also considered, without assigning numerical weights. Direct evidence from patients selected for anxiety or related psychological risk remains limited, mainly comprising small studies of pharmacological interventions, digital CBT, and selected adjunctive approaches. Although some studies reported improvements in anxiety, sleep, pain, or analgesic use, sustained benefits in function or satisfaction have not been consistently demonstrated (Table 2; Figure 2).

Studies conducted in general TKA populations without anxiety-based selection provide indirect evidence relevant to psychoeducational, cognitive behavioral, mind–body, digital, analgesic, and other adjunctive interventions. Although several interventions reduced anxiety scores, changes in anxiety, pain, and function did not consistently occur in parallel. Because anxiety was not an eligibility criterion, it remains uncertain whether patients with identified anxiety would derive similar benefits. These studies also cannot establish that reductions in anxiety mediated improvements in clinical outcomes (Table 3).

Evidence across intervention categories differs in applicability to anxiety-selected TKA patients, risk of bias, precision, and outcome consistency. Pharmacological studies provide some evidence from anxiety-selected populations, but small samples, mixed THA/TKA cohorts, and methodological limitations restrict interpretation. Psychoeducational and CBT studies mainly involve unselected patients, with heterogeneous interventions, missing data, and inconsistent long-term outcomes. Digital interventions also largely involve unselected populations, while the few studies targeting elevated psychological symptoms are small, with uncertain clinical relevance and durability. Adjunctive interventions primarily report short-term benefits, often with limitations related to blinding, attrition, or uncontrolled comparisons. Across these categories, small samples and incomplete effect estimates limit precision, while improvements in anxiety do not consistently translate into better pain, function, or satisfaction outcomes.

Accordingly, perioperative intervention studies should assess outcomes beyond anxiety scores, including pain, opioid use, rehabilitation participation, and long-term function, and determine whether observed changes are clinically meaningful. Study protocols, proof-of-concept trials, and secondary anxiety outcomes from general TKA populations should be interpreted separately from completed efficacy studies in anxiety-selected populations.

## 7. Limitations of the Current Evidence and Research Priorities

Across the 60 formally appraised studies, recurring methodological concerns included unclear allocation procedures, missing outcome data, residual confounding, and potential measurement bias (Supplementary Table S2). These limitations informed the interpretation of the evidence, with less emphasis placed on anxiety improvements affected by incomplete data or potentially biased measurements. Nonrandomized comparisons with inadequate confounding control were also considered insufficient to support strong causal conclusions.

### 7.1. Study Populations and Definitions of Anxiety

Existing studies have defined anxiety using scale-based thresholds, prior psychiatric diagnoses, or medication histories, and some have combined anxiety with depression, pain catastrophizing, or other psychiatric conditions. These approaches are not interchangeable and may identify different psychological constructs and levels of risk. Future studies should clearly distinguish state anxiety, trait anxiety, anxiety symptoms, and diagnosed anxiety disorders, with the assessment instrument, cutoff value, and handling of anxiety–depression comorbidity prespecified. In mixed THA/TKA populations, TKA-specific findings should be reported separately whenever possible.

### 7.2. Assessment Instruments and Timing of Measurement

HADS-A, GAD-7, STAI, and HAMA differ in the constructs assessed, mode of administration, and interpretation of scores and thresholds. Anxiety measured at different perioperative stages may also have different clinical implications. Future studies should therefore prespecify the primary assessment instrument and key measurement time points and report baseline values and the magnitude of change. Where validated and applicable, clinically important change thresholds such as the minimal clinically important difference (MCID) should also be reported. Statistical significance should be interpreted alongside clinical relevance, and selective reporting of favorable findings across multiple instruments or time points should be minimized.

### 7.3. Intervention Reporting and Adherence

Current interventions include pharmacological treatment, digital CBT, AI-assisted patient education, preoperative education, psychological and behavioral interventions, VR-based rehabilitation, music therapy, and acupuncture. Studies vary substantially in intervention content, timing, duration, frequency, and delivery. Future studies should report the theoretical rationale, core components, intervention dose, provider training, control conditions, co-interventions, and adherence. Single-component interventions should also be distinguished from multimodal programs. Digital and AI-based interventions require additional reporting on human oversight, information sources, system updates, privacy safeguards, and adverse events. More standardized reporting would improve reproducibility and facilitate interpretation across studies.

### 7.4. Core Outcomes and Follow-Up Time Points

Existing studies have assessed a wide range of outcomes, including anxiety, pain, range of motion, function, opioid use, satisfaction, and quality of life. However, primary endpoints, measurement instruments, and follow-up schedules remain heterogeneous, limiting between-study comparisons and quantitative synthesis. Future research should work toward a core outcome set that includes anxiety, acute and persistent pain, opioid use, patient-reported function, objective or performance-based function, satisfaction, quality of life, and adverse events. Key short-, intermediate-, and long-term assessment time points should be prespecified. Longer follow-up is particularly important to determine whether short-term improvements in anxiety are accompanied by sustained improvements in persistent pain and long-term function.

### 7.5. Need for High-Quality Direct Evidence

Few completed randomized controlled trials have specifically enrolled TKA patients on the basis of elevated anxiety or predefined psychological risk, and most available studies are small, single-center, or proof-of-concept investigations. Protocol-only studies cannot provide evidence of efficacy. Future multicenter trials should use prespecified and validated anxiety-screening criteria and clearly characterize coexisting depression, pain catastrophizing, and other relevant psychological or pain-related factors. Randomization and allocation procedures should be adequately described, outcome assessment should be blinded where feasible, and missing data, adverse events, and prespecified primary outcomes should be transparently reported. Where mechanistically justified, prespecified mediation analyses may test whether changes in anxiety are associated with subsequent changes in pain or rehabilitation behavior. Follow-up extending to 3, 6, and 12 months would help determine whether short-term psychological improvements are accompanied by sustained clinical benefits.

### 7.6. Limitations of This Review

This narrative review has several limitations. Study selection was not systematic, and quantitative synthesis was not undertaken because of heterogeneity in study populations, anxiety definitions, interventions, outcomes, and follow-up. Selection and publication bias therefore cannot be excluded, and the findings should be interpreted as a narrative synthesis rather than pooled estimates of treatment effects. Fuchs et al. [11] was retained for descriptive discussion but excluded from formal risk-of-bias appraisal because its allocation method could not be reliably classified. Its findings were therefore not considered evidence of VR superiority over conventional physiotherapy.

## 8. Implications for Clinical Management

### 8.1. Preoperative Screening and Risk Assessment

Anxiety assessment may be considered as one component of comprehensive preoperative evaluation in patients undergoing TKA. However, current evidence does not support a single screening instrument, universal cutoff value, or validated anxiety-based risk-stratification pathway. Brief validated scales may help identify patients with elevated anxiety symptoms. Their results should be interpreted together with depression, pain catastrophizing, kinesiophobia, sleep disturbance, and psychiatric history. Screening should not be used to establish a psychiatric diagnosis or to predict poor outcomes in isolation. A positive screening result should instead prompt further clinical assessment and, when appropriate, additional perioperative support. This may include individualized education, optimization of pain management, psychological support, or specialist referral. False-positive findings should also be considered, as they may lead to unnecessary concern, referral, or intervention.

### 8.2. Individualized Interventions

A uniform intervention approach is unlikely to be appropriate for all TKA patients with anxiety. As a pragmatic but currently unvalidated framework, patients with lower symptom burden may be offered clear preoperative education, expectation management, and rehabilitation guidance, whereas those with more prominent anxiety or concomitant kinesiophobia or pain catastrophizing may benefit from individualized communication, relaxation strategies, and tailored rehabilitation goals. Pain neuroscience education may be incorporated into broader educational programs, but current randomized evidence has not shown consistent benefits for anxiety or postoperative pain [67], and it should not be regarded as a stand-alone anxiety treatment. For patients with severe or persistent symptoms, comorbid depression, or substantial psychological complexity, further psychological or multidisciplinary assessment may be considered. The specific criteria for such stratification and the effectiveness of this approach require prospective validation.

### 8.3. Digital Health Support

Digital CBT, AI-assisted education, mobile rehabilitation, and remote follow-up may extend perioperative support across the preoperative, inpatient, and post-discharge periods. These tools may facilitate continuity of education, symptom monitoring, rehabilitation guidance, and communication, but should complement rather than replace clinical assessment and feedback. Target populations, intervention content, frequency and duration, and digital literacy requirements should be clearly defined. Conventional face-to-face options remain important, and digital interventions should account for access to technology, digital literacy, and the need for appropriate clinical oversight [21,71]. Evaluation should extend beyond satisfaction and engagement to clinically relevant outcomes, adherence, information quality, and, where validated and applicable, whether observed changes reach thresholds for clinical importance.

### 8.4. Multidisciplinary Management

Patients with clinically relevant anxiety may benefit from coordinated attention to psychological status, pain management, and rehabilitation. For those with severe, persistent, or clinically complex symptoms, multidisciplinary assessment may be considered, with input from orthopedics, anesthesiology or pain medicine, rehabilitation, nursing, psychiatry, or psychology as appropriate. Such collaboration may facilitate integration of expectation management, perioperative analgesia, rehabilitation goals, and psychological support across the surgical pathway. However, the optimal team structure, referral criteria, resource requirements, feasibility, and effectiveness of multidisciplinary management in routine TKA practice have not been established and require prospective evaluation.

## 9. Conclusions

Perioperative anxiety is common among patients undergoing TKA and is associated with greater postoperative pain, higher analgesic requirements, poorer patient-reported recovery, and lower satisfaction. It frequently coexists with other psychological and pain-related factors and should therefore be interpreted as part of a broader perioperative risk profile rather than as an isolated predictor. Some pharmacological, psychoeducational, cognitive behavioral, digital, and adjunctive interventions may reduce short-term anxiety symptoms, but these improvements do not consistently translate into sustained or clinically meaningful benefits in pain, opioid use, function, or satisfaction. Direct evidence from anxiety-screened TKA populations remains limited, particularly for long-term outcomes. Anxiety assessment may therefore be considered as one component of comprehensive preoperative evaluation, while validated assessment approaches and high-quality multicenter trials are needed to determine whether targeted interventions provide reproducible clinical benefits beyond improvements in anxiety itself.

## Figures and Tables

**Table 1:** Comparison of anxiety assessment instruments commonly used in the perioperative setting of total knee arthroplasty.

Instrument	Primary Construct Assessed	Administration	Score Range	Threshold and Purpose	Application in TKA
HADS-A [12,15]	General anxiety symptoms in patients with physical illness	7 items; patient self-report	0–21	≥8 is commonly used for screening	Suitable for screening and follow-up; limited specificity for surgery-related anxiety
GAD-7 [16]	Generalized anxiety symptoms during the previous 2 weeks	7 items; patient self-report	0–21	Scores of 5, 10, and 15 are commonly used to indicate mild, moderate, and severe symptom levels, respectively	Brief and suitable for repeated assessment; limited specificity for surgery-related anxiety
STAI [12]	State and trait anxiety	STAI-S and STAI-T each contain 20 items; patient self-report	20–80 for each full subscale	No universally accepted perioperative TKA cutoff	Distinguishes state from trait anxiety; useful for serial assessment but relatively lengthy and timing-sensitive
HAMA [17]	Severity of psychic and somatic anxiety	14 items; clinician-rated	0–56	No standardized perioperative cutoff	Suitable for further professional assessment of anxiety severity; relatively time-consuming and susceptible to the influence of pain and other somatic symptoms
APAIS [18]	Surgery- and anesthesia-related anxiety and need for information	6 items; patient self-report	Anxiety subscale: 4–20 (4 items); information need subscale: 2–10 (2 items)	An anxiety subscale score ≥ 11 is commonly used for preoperative screening	Specific to surgery and anesthesia; report anxiety and information need separately; unsuitable for trait anxiety or long-term anxiety assessment
Other instruments	SAS, PROMIS Anxiety, and VAS-A [19,20,21,22].	Varies by instrument	Varies by instrument	Defined according to the relevant validated version	Report the actual instrument, version, scoring method, and assessment time; measures are not interchangeable

Screening thresholds indicate symptom levels or possible anxiety; they are not diagnostic criteria or MCIDs. Use the validated language/version and report timing. The six-item APAIS total must not be interpreted as a pure anxiety score. HADS-A, Hospital Anxiety and Depression Scale anxiety subscale; GAD-7, Generalized Anxiety Disorder-7; STAI, State–Trait Anxiety Inventory; HAMA, Hamilton Anxiety Rating Scale; SAS, Self-Rating Anxiety Scale; PROMIS, Patient-Reported Outcomes Measurement Information System; VAS-A, Visual Analogue Scale for Anxiety. STAI-S, State Anxiety subscale of the State–Trait Anxiety Inventory; STAI-T, Trait Anxiety subscale of the State–Trait Anxiety Inventory.

**Table 2:** Completed perioperative intervention studies in populations selected using anxiety or anxiety/depression criteria and including patients undergoing TKA (direct evidence).

Study	Design and Study Population	Intervention	Key Findings	Directness and Outcome-Specific Appraisal
Yılmaz et al., 2015 [59]	Retrospective study; 76 TKA patients (38/group); HAMA ≥ 16 and BDI 10–23	Alprazolam plus standard analgesia vs. standard analgesia	Lower analgesic use over 5 postoperative days (4.25 ± 0.30 vs. 4.81 ± 0.41 g); improved knee function at 1 month	TKA-only, combined anxiety/depression eligibility. ROBINS-I: Serious risk for diclofenac use; nonrandom allocation and inadequate confounding control
Liang et al., 2017 [60]	Single-blind RCT; 61 TKA patients reported; HAMA > 16 and BDI 10–23	Alprazolam vs. placebo	Lower HAMA 1 day before surgery (13.1 ± 2.3 vs. 22.3 ± 4.2); lower postoperative pain, total NSAID use (1027 ± 182 vs. 1350 ± 217 mg) and analgesic use; no difference in knee-function improvement	RoB 2: Some concerns for preoperative HAMA. SAS-generated random codes reported; concealment and group counts unclear. Anxiety/depression not separated by eligibility
Jiang et al., 2026 [61]	Double-blind RCT; 102 randomized, 101 analyzed; mixed THA/TKA (61 TKA); STAI-S ≥ 40	Olanzapine vs. placebo	Lower perioperative anxiety (STAI-S Cohen’s d > 0.8) and moderate effects on major secondary outcomes (d > 0.5); no TKA functional benefit	RoB 2: Some concerns for anxiety. Pooled THA/TKA anxiety results limit TKA-specific directness; functional benefit confined to THA
Kaynar et al., 2023 [19]	Exploratory RCT; 15 TKA patients randomized, 12 completed; PROMIS anxiety/depression T-score ≥ 57; anxiety endpoint: 3/arm	Preoperative RxWell digital CBT vs. no intervention	PROMIS emotional scores decreased from 64.3 ± 3.0 to 58.5 ± 2.6 in the intervention group; no formal between-group effect size reported	RoB 2: High risk for preoperative anxiety; nonadherence exclusions, missing anxiety data, and combined anxiety/depression reporting
Kainkaryam et al., 2025 [72]	Open-label RCT; 60 randomized (31 THA/29 TKA); APAIS anxiety subscale > 10	Single acupuncture session on the day of surgery vs. no acupuncture	Within-group anxiety decreased from 60.73 ± 21.28 to 28.90 ± 21.54 (95% CI 23.56–40.10); no randomized between-group anxiety comparison	Mixed THA/TKA; anxiety change is uncontrolled, assessed separately with JBI quasi-experimental items. RoB 2: Some concerns apply to between-group PACU pain, not anxiety
Tailor et al., 2026 [22]	Pilot RCT; 30 TKA patients randomized, 24 assessed on POD7; PROMIS Anxiety Short Form raw score ≥ 19	Lavender–peppermint aromatherapy patch vs. almond oil placebo	No anxiety benefit at POD7 (PROMIS Anxiety MD −1.31, 95% CI −8.40 to 5.77)	RoB 2: High risk for POD7 anxiety, principally because of missing endpoint data; masking unsuccessful; short follow-up

APAIS, Amsterdam Preoperative Anxiety and Information Scale (four-item anxiety subscale); BDI, Beck Depression Inventory; CBT, cognitive behavioral therapy; HAMA, Hamilton Anxiety Rating Scale; JBI, Joanna Briggs Institute; LOS, length of stay; PACU, postanesthesia care unit; POD, postoperative day; PROMIS, Patient-Reported Outcomes Measurement Information System; RCT, randomized controlled trial; STAI-S, State Anxiety subscale of the State–Trait Anxiety Inventory; THA, total hip arthroplasty; TKA, total knee arthroplasty. Anxiety-based eligibility does not by itself establish direct TKA-specific evidence: mixed samples and uncontrolled anxiety changes are identified separately. RoB judgments apply only to the stated outcomes; the tools do not share a common quality scale. The Kaynar multicenter protocol [68] provides planned methods but no efficacy results and is not listed as a completed trial. Printed alprazolam doses in [59,60] require clarification and are not reproduced here.

**Table 3:** Representative indirect evidence for perioperative anxiety management in general TKA populations.

Study	Design and Study Population	Intervention	Key Findings	Outcome-Specific Appraisal and Limitation
Zheng et al., 2023 [62]	RCT; 208 TKA patients randomized, 161 analyzed	Multimodal analgesia vs. standard analgesia	Lower HADS anxiety scores on POD3 (10.7 ± 4.6 vs. 13.2 ± 5.3) and POD7 (9.2 ± 3.4 vs. 11.8 ± 4.7), but no between-group difference at 3 months (5.1 ± 1.8 vs. 5.5 ± 2.1); early pain and functional outcomes also favored multimodal analgesia	RoB 2: High risk for early HADS-A; substantial postrandomization exclusions. Anxiety improvement does not establish mediation of recovery
YaDeau et al., 2016 [64]	Triple-blind, placebo-controlled RCT; 106 TKA patients randomized	Duloxetine vs. placebo	Lower opioid consumption, but no HADS-A benefit at POD14 (median change −2 vs. −2; Wilcoxon–Mann–Whitney odds 1.14, 95% CI 0.73–1.81) and no ambulatory-pain benefit	RoB 2: Some concerns for POD14 HADS-A; anxiety secondary and baseline levels low
Medina-Garzón et al., 2019 [65]	RCT; knee arthroplasty; 56 enrolled, 55 analyzed	Motivational interviewing plus standard education vs. standard education	Lower postintervention APAIS scores before surgery	RoB 2: Some concerns. Reported APAIS scoring may include information need; no postoperative clinical outcomes
Hanley et al., 2021 [50]	Cluster-randomized trial; 47 clusters, 316 randomized, 285 included (181 TKA); 271 paired immediate assessments	A 15-min mindfulness or hypnotic suggestion intervention vs. cognitive behavioral education	Immediate anxiety reductions; better 6-week patient-reported function with mindfulness	Cluster RoB 2: Some concerns for immediate anxiety; High risk for 6-week function because of missing data. Mixed THA/TKA; recruitment timing and single-item anxiety limit interpretation
Baas et al., 2024 [67]	Single-center, parallel-group RCT; primary TKA; *n* = 68	Two individual sessions of pain neuroscience education (PNE) vs. usual care	No 3-month pain benefit (WOMAC pain mean difference 0.4 points, 95% CI −1.7 to 2.4) or HADS benefit (between-group difference −0.1, 95% CI −1.6 to 1.4); attention to pain was lower with PNE	RoB 2: Some concerns for 3-month HADS-A; small sample and variable intervention delivery
Reis and Candas Altinbas, 2026 [53]	Single-center RCT; older adults undergoing TKA; *n* = 120	Nurse-led structured preoperative education vs. standard education	Lower surgery-morning STAI-S (44.0 ± 4.9 vs. 50.4 ± 6.6, *p* < 0.001), with earlier mobilization and greater early activity; no postoperative pain or LOS benefit.	RoB 2: Some concerns for surgery-morning STAI-S; single-center, predominantly female sample; anxiety mediation not established
Chen et al., 2021 [20]	RCT; TKA patients aged ≥ 70 years; 90 randomized, 83 analyzed	Perioperative CBT vs. routine care	Lower SAS on PODs 7 and 14; no additional postoperative pain or knee-function improvement	RoB 2: High risk for early SAS; health-related and other missing outcome data. Anxiety and depression outcomes differ over time
Pester et al., 2023 [69]	Nonrandomized pilot; 25 enrolled, 22 intervention completers plus 22 matched historical controls	Brief mindfulness-based CBT vs. usual treatment	Lower pain severity/interference at 6 weeks, without sustained differences at 3 or 6 months	ROBINS-I: Serious risk for 6-week pain; historical controls and residual confounding. Proposed mediation concerns catastrophizing, not anxiety
Kara and Karabulut, 2026 [71]	Single-center RCT; unilateral TKA; 112 randomized, 108 analyzed	Mobile-delivered education, relaxation, and rehabilitation exercises vs. usual care through 4 weeks after discharge	Lower STAI-S through 4 weeks; at 4 weeks, the between-group mean difference was −7.15 points (95% CI −8.51 to −5.79; Cohen’s d = 1.996), but the study-specified 10-point MCID was not reached	RoB 2: Some concerns for week-4 STAI-S; multicomponent intervention, digital access requirement, short follow-up; no demonstrated anxiety mediation
Gan et al., 2025 [21]	Single-blind pilot RCT; 60 TKA patients randomized, 55 analyzed	Physician-supervised, ChatGPT 4.0-assisted informed consent vs. conventional informed consent	Lower HADS-A after informed consent (10.48 ± 3.84 vs. 12.75 ± 4.12) and on POD5 (8.33 ± 3.20 vs. 10.71 ± 3.83); satisfaction was higher, with no additional pain or knee-function benefit	RoB 2: Some concerns for postconsent HADS-A; High risk for POD5 HADS-A because of missing data. Five online supplements unavailable; sample accounting incomplete
Peuchot et al., 2021 [80]	Prospective nonrandomized cohort; 24 eligible, 20 TKA patients analyzed	Intraoperative VR distraction vs. routine spinal-anesthesia care	No next-day STAI-Y1 or satisfaction benefit; less sedation and greater comfort	ROBINS-I: Serious risk for next-day anxiety; preference-based allocation and uncontrolled confounding
Beşirik and Sayar, 2026 [81]	RCT; TKA under regional anesthesia; 70 randomized (35/group)	Intraoperative VR nature/landscape imagery vs. routine care	Lower immediate postsurgery STAI-S; some pulse-rate differences	RoB 2: Some concerns for immediate STAI-S; unblinded subjective assessment; longer-term outcomes untested
Wang et al., 2025 [82]	RCT; 116 TKA patients randomized; 114 in primary analysis, 109 with HADS observations	Digital CBT for sleep disorders vs. WeChat sleep education	Better early sleep/recovery scores and lower HADS-A on PODs 3 and 15; no additional pain benefit	RoB 2: Some concerns for HADS-A; anxiety secondary to a sleep-focused intervention; outcome denominators differ
Nalbant et al., 2026 [75]	Prospective, randomized, single-blind trial; TKA under spinal anesthesia; 80 randomized, 65 analyzed	Patient-preferred music before and during surgery vs. usual care	Lower postoperative STAI-S (36.09 ± 4.36 vs. 42.27 ± 6.21; between-group difference 6.18 points), exceeding the reported MCID of 2.18; the change-score difference of 3.92 points also exceeded the reported MCID of 3.37	RoB 2: High risk for postoperative STAI-S due to postrandomization exclusions and incomplete outcome data. No anxiety-based enrollment; single-center study with short-term outcomes.
Ursavas and Catakli, 2020 [76]	RCT; TKA; 38 randomized (19/group)	Diaphragmatic breathing exercises vs. routine care	Lower HADS-A on POD2; no pain or depression benefit	RoB 2: Some concerns for POD2 HADS-A; concealment and prospective analysis specification insufficiently documented
Dağci and Deniz Öztekin, 2025 [77]	Four-arm RCT; TKA; 100 randomized (25/group)	Active noise cancellation, passive noise control, or active noise cancellation with music vs. no device	Lower 24 h STAI-I with passive noise control and noise cancellation with music; lower pain in all intervention arms	RoB 2: Some concerns for 24-h STAI-I; concealment/analysis specification unclear. Combined noise cancellation/music does not isolate a music effect
Kaplun et al., 2026 [78]	RCT; first elective TKA; 52 randomized, 42 analyzed	Brief daily guided imagery plus standard care vs. standard care for up to 6 weeks	No between-group anxiety or WOMAC benefit; some pain and functional-rating outcomes improved	RoB 2: High risk for state anxiety; attrition and a reported mean below the stated STAI scale minimum impair interpretation
Tanriver et al., 2026 [79]	RCT; TKA; 112 randomized, 80 analyzed	Emotional freedom technique on POD2 vs. routine care	Lower immediate postintervention STAI-S and pain on POD2	RoB 2: High risk for immediate STAI-S; substantial health/adherence-related exclusions and unblinded investigator-administered measurement

APAIS, Amsterdam Preoperative Anxiety and Information Scale; CBT, cognitive behavioral therapy; HADS-A, Hospital Anxiety and Depression Scale anxiety subscale; LOS, length of stay; MCID, minimal clinically important difference; PNE, pain neuroscience education; POD, postoperative day; QoR-15, 15-item Quality of Recovery questionnaire; RCT, randomized controlled trial; SAS, Self-Rating Anxiety Scale; STAI-S, State Anxiety subscale of the State–Trait Anxiety Inventory; THA, total hip arthroplasty; TKA, total knee arthroplasty; VR, virtual reality; WOMAC, Western Ontario and McMaster Universities Osteoarthritis Index. This table presents representative rather than exhaustive evidence. Randomized and analyzed sample sizes are distinguished where available. RoB 2 and ROBINS-I judgments are outcome-specific and are not interchangeable. Statistical differences do not establish clinical importance or anxiety-mediated recovery. The Lloyd systematic review [66] is summarized separately in the text and is not counted as a primary intervention study. Fuchs [11] is not given a formal RoB conclusion because allocation remains unresolved.

## Data Availability

No new data were created or analyzed in this study. Data sharing is not applicable to this article.

## Supplementary Materials

The following supporting information can be downloaded at: https://www.mdpi.com/article/10.3390/jcm15208140/s1, Table S1: Database-specific literature search strategies used for the narrative review; Table S2: Summary risk-of-bias assessment of the 60 formally appraised studies.
