# Supplementary material for: Perioperative Anxiety in Total Knee Arthroplasty: Clinical Consequences, Potential Mechanisms, and Management—A Narrative Review
Source: J Clin Med. 2026 Oct 20;15(20):8140. doi: 10.3390/jcm15208140 (PMC13706237; doi:10.3390/jcm15208140)
Supplement: Supplementary file 1 [file jcm-15-08140-s001.zip › jcm-4568633-supplementary.pdf]

Supplementary Table S1. Database-specific literature search strategies used for the narrative review.
Supplementary Table S2. Summary risk-of-bias assessment of the 60 formally appraised studies

Supplementary Table S1. Database-specific literature search strategies used for the narrative review.

Database	Search strategy	Date and language restriction	Final search date	Records of Articles
PubMed (National Library of Medicine)	("Arthroplasty, Replacement, Knee"[Mesh] OR "total knee arthroplasty"[Title/Abstract]<br>OR "total knee replacement"[Title/Abstract] OR "knee arthroplasty"[Title/Abstract]<br>OR "knee replacement"[Title/Abstract]<br>OR TKA[Title/Abstract]<br>OR TKR[Title/Abstract])<br>AND<br>(Anxiety[Mesh]<br>OR anxi*[Title/Abstract]◎OR "perioperative anxiety"[Title/Abstract]◎OR "preoperative anxiety"[Title/Abstract]◎OR "postoperative anxiety"[Title/Abstract] OR "state anxiety"[Title/Abstract] OR "trait anxiety"[Title/Abstract])	No date and language restriction	25 September 2026	716
Embase (Elsevier Embase.com)	('knee arthroplasty'/exp<br>OR 'knee arthroplasty'<br>OR 'total knee arthroplasty':ti,ab,kw<br>OR 'total knee replacement':ti,ab,kw<br>OR 'knee arthroplasty':ti,ab,kw<br>OR 'knee replacement':ti,ab,kw<br>OR tka:ti,ab,kw<br>OR tkr:ti,ab,kw)<br>AND<br>('anxiety'/exp<br>OR 'anxiety'<br>OR anxi*:ti,ab,kw)	No date and language restriction	25 September 2026	1268
Web of Science (Clarivate)	ALL=("total knee arthroplasty"<br>OR "total knee replacement"<br>OR "knee arthroplasty"<br>OR "knee replacement"<br>OR TKA<br>OR TKR)<br>AND<br>ALL=(anxi*)	No date and language restriction	25 September 2026	926

Database	Search strategy	Date and language restriction	Final search date	Records of Articles
China National Knowledge Infrastructure (CNKI; Subject)	(全膝关节置换 OR 全膝置换 OR 人工全膝关节置换 OR 膝关节置换 OR TKA OR TKR) AND (焦虑 OR 术前焦虑 OR 术后焦虑 OR 围术期焦虑 OR 围手术期焦虑) English translation (for reference): (total knee arthroplasty OR total knee replacement OR artificial total knee arthroplasty OR knee arthroplasty OR TKA OR TKR) AND (anxiety OR preoperative anxiety OR postoperative anxiety OR perioperative anxiety)	No date and language restriction	25 September 2026	231

The literature search was conducted in PubMed, Embase, Web of Science Core Collection, and China National Knowledge Infrastructure (CNKI). No date restriction or language restriction was applied. Database-specific controlled vocabulary, subject headings, and free-text terms were combined to capture studies related to total knee arthroplasty and perioperative anxiety. The final search was conducted on 25 September 2026. The CNKI search strategy is presented using the original Chinese search terms, with an English translation provided for reference. Abbreviations: CNKI, China National Knowledge Infrastructure; TKA, total knee arthroplasty; TKR, total knee replacement.

Supplementary Table S2. Summary risk-of-bias assessment of the 60 formally appraised studies.

Study	Study design	Selected outcome/result	Appraisal tool	Risk-of-bias judgement	Main methodological concern(s)	Domain-level judgments and supporting rationale
RoB 2 (n = 23)						
Hanley 2021	Cluster-randomized trial	Immediate single-item anxiety: mindfulness / hypnotic suggestion versus CBT education<br>Additional: PROMIS physical function at 6 weeks: mindfulness/hypnotic suggestion versus CBT education	RoB 2 cluster	Immediate anxiety: Some concerns<br>6-week function: High risk	Uncertain allocation/recruitment safeguards; immediate missing-data concerns; self-report and unverified analysis plan. Greater attrition at 6 weeks.	Anxiety: D1 SC, D2 Low, D3 SC, D4 SC, D5 SC, D1b SC<br>6-week function: D1 SC, D1b SC, D2 Low, D3 High, D4 SC, D5 SC<br>Immediate anxiety: 47 clusters were randomized, but sequence implementation and postallocation recruitment safeguards were insufficiently documented (D1/D1b); 316 randomized, 285 included and 271 immediate paired assessments (D3). The unblinded, single-item self-report outcome and unavailable prespecified analysis plan contribute D4/D5 concerns. At 6 weeks, functional outcome data were available for only about 74% of participants (D3 High).
Reis 2026	Parallel randomized trial	Surgery-morning STAI-S: nurse-led preoperative education versus usual care	RoB 2	Some concerns	Incomplete concealment details; open-label self-report; unverified outcome-specific analysis plan.	D1 SC, D2 Low, D3 Low, D4 SC, D5 SC<br>Random-sequence and allocation-concealment details were incomplete (D1). Participants knew their education assignment while reporting STAI-S (D4), and the prospective analysis plan for this anxiety result could not be verified (D5).
Liang 2017	Placebo-controlled parallel trial reporting randomization; reporting inconsistencies	HAMA 1 day before surgery versus 2-week baseline: alprazolam versus placebo	RoB 2	Some concerns	Uncertain concealment/assessor masking; inconsistent sample accounting; incomplete deviation reporting; unverified analysis plan.	D1 SC, D2 SC, D3 SC, D4 SC, D5 SC<br>SAS-generated random codes were reported, but concealment was insufficiently documented and the reported 61 participants did not reconcile with 31 per group (D1/D3). Staff masking and deviations were unclear (D2); assessor masking for HAMA and the prespecified analysis plan were not verifiable (D4/D5).

Study	Study design	Selected outcome/result	Appraisal tool	Risk-of-bias judgement	Main methodological concern(s)	Domain-level judgments and supporting rationale
Jiang 2026	Double-blind parallel randomized trial	STAI-S before surgery / POD1 / POD3: olanzapine versus placebo	RoB 2	Some concerns	Outcome-specific analysis plan not independently available before unblinding.	D1 Low, D2 Low, D3 Low, D4 Low, D5 SC<br>The double-blind trial reported the STAI-S assessments, but a pre-unblinding analysis plan specifying these anxiety results was not independently available (D5). Other appraised domains were rated Low risk.
Zheng 2023	Parallel randomized trial	HADS-A POD3/7: multimodal analgesia versus routine analgesia	RoB 2	High risk	Substantial post-randomization losses; contradictory exclusion accounting; missingness likely related to psychological outcomes.	D1 SC, D2 Low, D3 High, D4 SC, D5 SC<br>Of 208 randomized patients, only 161 were analyzed; the flowchart and text gave inconsistent exclusion counts. Psychological or health-related exclusions could depend on underlying anxiety (D3 High). Allocation concealment, self-report measurement and prespecified analysis were insufficiently documented (D1/D4/D5).
Sarridou 2016	Double-blind parallel randomized trial	Reported STAI2 endpoint at 36–48 hours: parecoxib versus saline placebo	RoB 2	Some concerns	Inconsistent state/trait labels and retrospective measurement timing; unverified selected-result analysis plan.	D1 Low, D2 Low, D3 Low, D4 SC, D5 SC<br>Inconsistent STAI state/trait labels and retrospective timing of preoperative assessments leave the selected anxiety measure uncertain (D4). The prespecified STAI2 analysis was not independently verified (D5).
YaDeau 2016	Triple-blind parallel randomized trial	HADS-A change at postoperative day 14: duloxetine versus placebo	RoB 2	Some concerns	Pre-unblinding analysis plan for the secondary anxiety result not independently verified.	D1 Low, D2 Low, D3 Low, D4 Low, D5 SC<br>POD14 HADS-A was a secondary anxiety endpoint. Its pre-unblinding analysis plan could not be independently verified (D5); the other appraised domains were Low risk.

Study	Study design	Selected outcome/result	Appraisal tool	Risk-of-bias judgement	Main methodological concern(s)	Domain-level judgments and supporting rationale
Medina-Garzón 2019	Parallel randomized trial	Post-intervention APAIS before surgery: motivational interviewing versus routine care	RoB 2	Some concerns	Incomplete concealment safeguards; identifiable intervention and self-report; unverified prospective result selection.	D1 SC, D2 Low, D3 Low, D4 SC, D5 SC<br>Computer-generated assignment was described, but safeguards for concealing the printed sequence were incomplete (D1). Identifiable motivational interviewing and self-reported APAIS, potentially including information needs, affect outcome interpretation (D4). Prospective analysis specification was unavailable (D5).
Baas 2024	Parallel randomized trial	HADS-A at 3 months: preoperative pain neuroscience education versus usual care	RoB 2	Some concerns	Open-label self-report; unverified pre-unblinding analysis plan for secondary anxiety.	D1 Low, D2 Low, D3 Low, D4 SC, D5 SC<br>Open-label education may influence self-reported HADS-A (D4). The prespecified analysis for the secondary 3-month anxiety outcome was not independently available (D5).
Kaynar 2023	Pilot parallel randomized trial	Preoperative PROMIS anxiety: RxWell versus no intervention	RoB 2	High risk	Non-adherence exclusions; extensive anxiety-endpoint missingness; pooled anxiety/depression scores.	D1 SC, D2 High, D3 High, D4 High, D5 SC<br>Fifteen participants were randomized, but anxiety-endpoint data were available for only three per group; nonparticipation/nonadherence exclusions compromised an assignment-effect analysis (D2/D3 High). Anxiety and depression PROMIS outcomes were partly pooled, limiting anxiety-specific measurement (D4 High).
Kara 2026	Parallel randomized trial	STAI-S at postoperative week 4: mobile-based training versus routine care	RoB 2	Some concerns	Incomplete concealment safeguards; exclusions from assignment-effect analysis; open-label self-report; unverified analysis plan.	D1 SC, D2 SC, D3 Low, D4 SC, D5 SC<br>Allocation concealment was insufficiently documented (D1), and four of 112 randomized participants were excluded from the assignment-effect analysis (D2). An unblinded mobile intervention, self-reported STAI-S and unavailable prespecified analysis plan explain D4/D5 concerns.

Study	Study design	Selected outcome/result	Appraisal tool	Risk-of-bias judgement	Main methodological concern(s)	Domain-level judgments and supporting rationale
Gan 2025	Pilot parallel randomized trial	HADS-A immediately after preoperative consent: ChatGPT-assisted versus conventional consent Additional: HADS-A POD5: ChatGPT-assisted versus conventional consent	RoB 2	Immediate HADS-A: Some concerns POD5 HADS-A: High risk	Incomplete concealment/sample accounting; identifiable consent mode; unavailable analysis-plan materials. POD5 results vulnerable to wound-related exclusions.	Postconsent HADS-A: D1 SC, D2 Low, D3 SC, D4 SC, D5 SC<br>POD5 HADS-A: D1 SC, D2 Low, D3 High, D4 SC, D5 SC<br>Sixty patients were randomized and 55 analyzed. Wound-related postoperative exclusions were less clearly related to the immediate postconsent outcome (D3 Some concerns) but could affect POD5 anxiety (D3 High). Participants could identify the consent mode, and five online supplements/prespecified analyses were unavailable (D4/D5).
Kainkaryam 2025	Parallel randomized trial plus uncontrolled anxiety pre/post substudy	Between-group PACU pain at 1/3 hours. Additional: VAS-A before/after acupuncture; no post-intervention anxiety control.	RoB 2 (pain); JBI quasi (anxiety)	Pain: Some concerns Anxiety (JBI): J4: No; J5: No	Incomplete concealment; open-label pain self-report; unverified analysis plan. Anxiety has no post-intervention control or repeated pre/post series.	PACU pain: D1 SC, D2 Low, D3 Low, D4 SC, D5 SC<br>Anxiety (JBI quasi): J4 No, J5 No<br>The randomized comparison supported assessment of PACU pain only; concealment and open-label subjective pain measurement were incompletely safeguarded (RoB 2 D1/D4). Postintervention anxiety was measured only in acupuncture recipients, with no concurrent control-group anxiety measurement or repeated pre/post series (JBI J4/J5 No).
Tailor 2026	Participant-blinded pilot randomized trial; masking unsuccessful	POD7 STAI short form/PROMIS Anxiety SF: lavender-peppermint aromatherapy versus almond-oil control	RoB 2	High risk	Incompletely explained POD7 missingness; unsuccessful masking may influence self-report; unverified detailed analysis plan.	D1 Low, D2 Low, D3 High, D4 SC, D5 SC<br>Only 24 of 30 randomized participants provided POD7 questionnaires, with incomplete explanations of missingness (D3 High). Participant masking was largely unsuccessful and could influence self-reported anxiety (D4); the detailed prospective analysis plan was unavailable (D5).

Study	Study design	Selected outcome/result	Appraisal tool	Risk-of-bias judgement	Main methodological concern(s)	Domain-level judgments and supporting rationale
Abdul Hamid 2022	Parallel randomized trial	Short-term postoperative STAI-S: music versus no music	RoB 2	Some concerns	Incomplete envelope safeguards; identifiable music intervention and self-report; unverified analysis plan.	D1 SC, D2 Low, D3 Low, D4 SC, D5 SC<br>Computer randomization and sealed envelopes were reported, but envelope concealment safeguards were incomplete (D1). Participants could identify whether music was delivered (D4), and a prospective anxiety analysis plan was not verified (D5).
Nalbant 2026	Parallel randomized trial	STAI-S 4–6 hours after surgery: music versus inactive-headphone control	RoB 2	High risk	Exclusions for sedation, inadequate anesthesia or incomplete questionnaires threaten assignment-effect analysis and outcome-data completeness.	D1 SC, D2 High, D3 High, D4 SC, D5 SC<br>Excluding patients after randomization for sedation, insufficient spinal anesthesia or incomplete questionnaires compromised assignment-based analysis and completeness (D2/D3 High; 80 randomized, 65 analyzed). Music could remain identifiable despite headphones (D4), and prospective STAI-S analysis was not verified (D5).
Chen 2021	Parallel randomized trial	SAS at POD7/14: cognitive behavioral therapy versus routine care	RoB 2	High risk	Health-related/other losses with uncertain missingness mechanism; limited deviation reporting; unverified analysis plan.	D1 SC, D2 SC, D3 High, D4 SC, D5 SC<br>Ninety patients were randomized and 83 analyzed; ICU admission, falls and loss to follow-up left anxiety-outcome missingness uncertain (D3 High). Deviation reporting was incomplete (D2), and unblinded SAS self-report and unverified prespecification affected D4/D5.
Beşirik 2026	Parallel randomized trial	Immediate perioperative STAI-S: VR versus routine care	RoB 2	Some concerns	Identifiable VR intervention and subjective measurement; unverified prospective anxiety analysis plan.	D1 Low, D2 Low, D3 Low, D4 SC, D5 SC<br>VR delivery was apparent to participants, so immediate STAI-S self-report might be influenced by intervention knowledge (D4); prospective anxiety analysis specification was not independently available (D5).

Study	Study design	Selected outcome/result	Appraisal tool	Risk-of-bias judgement	Main methodological concern(s)	Domain-level judgments and supporting rationale
Kaplun 2026	Parallel randomized trial	STAI state-anxiety trajectory to 6 weeks: guided imagery versus control	RoB 2	High risk	Outcome missingness and unclear denominators; reported STAI mean below the stated scale minimum.	D1 Low, D2 Low, D3 High, D4 High, D5 SC<br>Only 42 of 52 randomized participants were analyzed, with incomplete anxiety denominators (D3 High). A reported STAI mean of 16.65 fell below the stated minimum score of 20, undermining interpretation of the anxiety measure (D4 High).
Tanriver 2026	Parallel randomized trial	STAI-S immediately after POD2 EFT: EFT versus routine care	RoB 2	High risk	Substantial health/adherence-related exclusions; immediate investigator-administered anxiety reporting susceptible to expectancy/social-desirability effects.	D1 SC, D2 High, D3 High, D4 High, D5 SC<br>Thirty-two of 112 randomized participants were excluded for health, withdrawal or adherence reasons (D2/D3 High). Immediate, investigator-administered anxiety assessment after EFT may be influenced by expectancy/social-desirability responses (D4 High); analysis prespecification was not verified (D5).
Ursavas 2020	Parallel randomized trial	HADS-A on POD2: deep breathing exercises versus routine care	RoB 2	Some concerns	Incomplete concealment and implementation details; identifiable intervention and self-report; unverified analysis plan.	D1 SC, D2 Low, D3 Low, D4 SC, D5 SC<br>Block randomization and coin tossing were described, but allocation concealment was not fully reported (D1). Breathing exercises were identifiable during HADS-A self-report (D4), and the prespecified analysis was not independently available (D5).
Dağci 2025	Parallel four-arm randomized trial	STAI-I at 24 hours: active noise cancellation, passive noise control or noise cancellation with music versus no-device control	RoB 2	Some concerns	Incomplete concealment safeguards; identifiable intervention and self-report; unverified prospective analysis specification.	D1 SC, D2 Low, D3 Low, D4 SC, D5 SC<br>An independently prepared computer random list was reported, but implementation/concealment safeguards were incomplete (D1). Intervention modes were identifiable during STAI self-report (D4); prospective multiarm anxiety analysis specifications were not verifiable (D5).

Study	Study design	Selected outcome/result	Appraisal tool	Risk-of-bias judgement	Main methodological concern(s)	Domain-level judgments and supporting rationale
Wang 2025	Parallel randomized trial	HADS-A on POD3/15: digital CBT versus WeChat sleep education	RoB 2	Some concerns	Non-adherence exclusions and differing HADS/primary-analysis denominators; open-label self-report; unverified analysis plan.	D1 SC, D2 SC, D3 SC, D4 SC, D5 SC<br>Of 116 randomized patients, 114 entered the primary analysis and only 109 contributed HADS data; the latter denominator differed from the primary analysis (D2/D3). The open-label sleep intervention and unverified analysis plan raise D4/D5 concerns.
ROBINS-I (n = 4)						
Yılmaz 2015	Retrospective non-randomized comparative intervention study	Alprazolam versus observation; recorded diclofenac use over 5 postoperative days	ROBINS-I (2016)	Serious risk	Non-random allocation and inadequate baseline adjustment; insufficient participant-selection and missing-data information.	I1 Serious, I2 NI, I3 Low, I4 Low, I5 NI, I6 Low, I7 Moderate<br>Patients were assigned to alprazolam or observation nonrandomly, without adequate adjustment for baseline anxiety, pain, function or psychiatric comorbidity (I1 Serious). Retrospective selection and missing-outcome denominators were insufficiently reported (I2/I5 No information).
Mi 2025	Prospective non-randomized matched comparative intervention cohort	Preoperative PAS-7 at T2/T3: auricular plaster versus no plaster, TKA-stratified	ROBINS-I (2016)	Serious risk	Patient choice and unmeasured true pretreatment anxiety; selection after completion; unblinded subjective outcomes.	I1 Serious, I2 Serious, I3 Low, I4 Low, I5 Low, I6 Serious, I7 Moderate<br>Auricular-plaster use was chosen by patients; propensity-score matching did not adjust for true pretreatment anxiety or treatment preferences (I1 Serious). Selection depended on intervention completion, with inconsistent sample accounting (I2 Serious); unblinded self-reported PAS-7 supports I6 Serious.

Study	Study design	Selected outcome/result	Appraisal tool	Risk-of-bias judgement	Main methodological concern(s)	Domain-level judgments and supporting rationale
Peuchot 2021	Prospective non-randomized comparative intervention study	Next-day STAI-Y1: intraoperative VR distraction versus routine spinal-anesthesia care	ROBINS-I (2016)	Serious risk	Preference-based allocation and unadjusted group differences; uncertain participant selection; unblinded STAI self-report.	I1 Serious, I2 NI, I3 Low, I4 Low, I5 Low, I6 Serious, I7 Moderate<br>VR exposure depended on patient preference and baseline differences were not adequately adjusted (I1 Serious). Four of 24 eligible patients did not provide data, with unclear timing of their exclusion (I2 No information); the unblinded patient-reported STAI endpoint supports I6 Serious.
Pester 2023	Non-randomized prospective intervention with historical controls	BPI pain severity at 6 weeks: perioperative MBCBT versus usual treatment	ROBINS-I (2016)	Serious risk	Historical controls and insufficient baseline/era confounding control; unblinded subjective pain assessment.	I1 Serious, I2 Low, I3 Low, I4 Low, I5 Low, I6 Serious, I7 Moderate<br>The intervention group was compared with historical controls from an earlier care period; demographic matching did not control baseline pain, treatment/era effects or motivation (I1 Serious). Unblinded self-reported BPI pain supports I6 Serious.
QUIPS (n = 23)						
Xu 2020	Prospective prognostic cohort	Preoperative HADS-A association with WOMAC at 6/18 weeks and 1 year	QUIPS	Q1: M; Q2: M; Q3: M; Q4: L; Q5: H; Q6: M	Insufficient confounding adjustment; inconsistent HADS item description; incomplete follow-up accounting.	The Methods described an HADS item structure inconsistent with the standard instrument (Q3 Moderate). Correlations between preoperative anxiety and WOMAC lacked adequate adjustment for baseline function, depression and other predictors (Q5 High); follow-up denominators were incompletely reported (Q2 Moderate).

Study	Study design	Selected outcome/result	Appraisal tool	Risk-of-bias judgement	Main methodological concern(s)	Domain-level judgments and supporting rationale
Zalikha 2021	Retrospective administrative prognostic cohort	Recorded anxiety diagnosis and index-admission complications / resource use, TKA stratum	QUIPS	Q1: L; Q2: L; Q3: M; Q4: M; Q5: M; Q6: L	Possible administrative-code misclassification; unmeasured anxiety severity, baseline pain and psychiatric medication use.	Exposure was identified through administrative diagnosis codes, which may misclassify anxiety (Q3 Moderate). Anxiety severity, baseline pain and psychiatric medication exposure were unavailable for full control (Q5 Moderate).
Martin 2024	Prospective cohort, secondary exploratory clustering analysis	Preoperative psychological / sensitization clusters and 6-month KOOS-JR/opioid use	QUIPS	Q1: M; Q2: M; Q3: M; Q4: L; Q5: M; Q6: M	Exploratory cluster construction without external validation; incomplete attrition information; residual confounding.	Psychological/sensitization clusters were constructed exploratorily without external validation (Q6 Moderate). Attrition details and residual confounding were incompletely addressed (Q2/Q5 Moderate).
Paredes 2024	Prospective longitudinal prognostic cohort	Preoperative HADS-A and pain at 48 hours; TKA-specific longitudinal association	QUIPS	Q1: M; Q2: M; Q3: L; Q4: L; Q5: M; Q6: M	Complete-case deletion; uncertain missingness; residual confounding and model robustness.	Longitudinal analyses were based on complete cases, and missingness assumptions were not fully tested (Q2 Moderate). Confounding and model robustness may affect anxiety–pain associations (Q5/Q6 Moderate).
Croppi 2025	Prospective prognostic cohort	Preoperative STAI trait anxiety and chronic pain at 6 months (VAS ≥4/10)	QUIPS	Q1: M; Q2: M; Q3: L; Q4: L; Q5: H; Q6: H	Inconsistent control of baseline pain/function; data-driven predictor selection and ROC cut-offs; risk of overfitting.	Baseline pain and function were not consistently accounted for in predictor analyses (Q5 High). Data-driven predictor selection and ROC cut-offs were assessed without independent validation, increasing overfitting risk (Q6 High).
Varış 2023	Prospective prognostic cohort	Preoperative STAI-S/T groups and VAS/Lysholm at 3 and 6 months	QUIPS	Q1: M; Q2: M; Q3: L; Q4: L; Q5: H; Q6: M	Unadjusted, dichotomized anxiety comparisons; incomplete follow-up accounting; multiple testing.	Anxiety groups were dichotomized and compared without sufficient covariate adjustment (Q5 High). Follow-up accounting and multiple testing across outcomes/time points were incompletely addressed (Q2/Q6 Moderate).

Study	Study design	Selected outcome/result	Appraisal tool	Risk-of-bias judgement	Main methodological concern(s)	Domain-level judgments and supporting rationale
Rivas 2025	Retrospective prognostic cohort	Recorded preoperative anxiety diagnosis and discharge pain/inpatient opioid use	QUIPS	Q1: M; Q2: L; Q3: M; Q4: L; Q5: M; Q6: M	Unverified recorded diagnoses; insufficient control of baseline pain, opioid exposure and anxiety treatment.	Recorded anxiety diagnoses could not be independently verified (Q3 Moderate). Baseline pain, preoperative opioid exposure and anxiety treatment were not sufficiently controlled (Q5 Moderate).
Rice 2018	Prospective prognostic cohort	Preoperative trait anxiety and moderate/severe persistent pain at 6/12 months	QUIPS	Q1: M; Q2: L; Q3: L; Q4: L; Q5: M; Q6: H	Extensive data-driven model selection without independent validation; residual confounding.	Extensive data-driven predictor selection was not independently validated (Q6 High). Residual confounding of the anxiety–persistent pain association remained possible (Q5 Moderate).
Lopez-Olivo 2020	Prospective prognostic cohort with timing ambiguity	DASS-21 / psychosocial variables and 24-month WOMAC / SF-36 / satisfaction	QUIPS	Q1: M; Q2: H; Q3: M; Q4: L; Q5: M; Q6: H	Inconsistent exposure timing; differential attrition; stepwise models with insufficient robustness checks.	Psychosocial exposure timing was inconsistently described (Q3 Moderate). Differential attrition and stepwise models without adequate robustness checks support Q2/Q6 High.
Heijbel 2024	Retrospective registry prognostic analysis	EQ-5D anxiety / depression item and 1-year dissatisfaction among improvement responders	QUIPS	Q1: M; Q2: H; Q3: M; Q4: L; Q5: M; Q6: M	Selection through PROM completeness and responder restriction; combined anxiety/depression item; residual confounding.	Inclusion depended on complete patient-reported outcome data and on having improved after surgery (Q2 High). Anxiety and depression were combined in one item, limiting construct specificity (Q3 Moderate).
Jia 2026	Retrospective prognostic cohort	Psychiatric diagnoses and 1-year KOOS improvement / PASS / satisfaction	QUIPS	Q1: M; Q2: M; Q3: M; Q4: L; Q5: M; Q6: M	Missingness assumptions despite multiple imputation; heterogeneous recorded psychiatric diagnoses; unclear repeated-patient handling.	Although multiple imputation was used, missingness assumptions remained uncertain (Q2 Moderate). Recorded psychiatric conditions were heterogeneous and repeated-patient handling was unclear (Q3/Q6 Moderate).

Study	Study design	Selected outcome/result	Appraisal tool	Risk-of-bias judgement	Main methodological concern(s)	Domain-level judgments and supporting rationale
Aalders 2025	Prospective prognostic cohort	Preoperative HADS-A and pain / function / MCID / PASS up to 2 years	QUIPS	Q1: M; Q2: H; Q3: L; Q4: L; Q5: M; Q6: M	Exclusion for incomplete long-term follow-up; residual clinical/psychological confounding.	Patients without complete long-term follow-up were excluded, potentially selecting outcomes or psychological profiles (Q2 High). Residual clinical and psychological confounding remained possible (Q5 Moderate).
Ali 2017	Prospective prognostic follow-up of trial participants	Preoperative HADS anxiety / depression symptoms and 4-year dissatisfaction	QUIPS	Q1: M; Q2: L; Q3: L; Q4: L; Q5: M; Q6: M	Incomplete control of baseline pain and psychological comorbidity; combined anxiety/depression interpretation.	Anxiety and depression were partly interpreted together, and baseline pain or comorbid psychological factors were not sufficiently accounted for (Q5 Moderate).
Carriere 2022	Prospective prognostic cohort	Preoperative pain/function expectations and postoperative WOMAC at 6 weeks/6 months/1 year	QUIPS	Q1: M; Q2: H; Q3: M; Q4: L; Q5: L; Q6: M	Follow-up-dependent inclusion and complete-case missingness; limited independent validation of expectation items.	Eligibility for some analyses depended on completing follow-up and complete-case data (Q2 High). Expectation items were not independently validated (Q3 Moderate).
Fillingham 2021	Retrospective prognostic cohort	Preoperative GAD-2/treatment status and 6-week Pain Catastrophizing Scale	QUIPS	Q1: M; Q2: H; Q3: M; Q4: L; Q5: H; Q6: M	Large exclusions; insufficiently documented adjustment; baseline pain, depression, opioid use and treatment-indication confounding.	Large exclusions raised attrition concerns (Q2 High). Baseline pain, depression, opioids and confounding by treatment indication were incompletely controlled (Q5 High).
Bierke 2017	Prospective prognostic cohort	Preoperative anxiety / catastrophizing and pain / KOOS / satisfaction at 6/12 months	QUIPS	Q1: M; Q2: H; Q3: L; Q4: L; Q5: H; Q6: M	Substantial attrition; insufficient adjustment for baseline clinical and psychological factors.	Substantial follow-up attrition (Q2 High) was combined with limited adjustment for baseline clinical/psychological predictors of pain and satisfaction (Q5 High).

Study	Study design	Selected outcome/result	Appraisal tool	Risk-of-bias judgement	Main methodological concern(s)	Domain-level judgments and supporting rationale
Wylde 2012	Prospective prognostic cohort	Preoperative self-efficacy / anxiety and 1-year WOMAC pain/function	QUIPS	Q1: M; Q2: M; Q3: L; Q4: L; Q5: L; Q6: L	Participant-selection and follow-up assumptions remain uncertain despite extensive clinical adjustment.	Study participation and follow-up assumptions were not fully established (Q1/Q2 Moderate), although multivariable clinical adjustment supported a Low-risk Q5 judgment.
Bruehl 2024	Prospective cohort, secondary mediation analysis	Preoperative trait anxiety–oxidative stress–6-month pain/interference association	QUIPS	Q1: M; Q2: M; Q3: L; Q4: L; Q5: H; Q6: M	Baseline pain omitted from mediation adjustment; complete-case selection; exploratory mediation estimates.	Baseline pain was omitted from the mediation adjustment, and complete-case selection may affect exploratory estimates (Q5 High; Q2 Moderate). The results do not independently establish a causal mediating pathway.
Aalders 2026	Prospective prognostic cohort	Preoperative HADS-A and objective physical performance at 12 months	QUIPS	Q1: M; Q2: H; Q3: L; Q4: L; Q5: M; Q6: L	Substantial attrition and motivation-related selection; covariate adjustment differs across models.	Long-term attrition and motivation-related selection support Q2 High; covariate sets differed across physical performance models (Q5 Moderate).
Hiyama 2019	Prospective prognostic cohort	Discharge context-specific anxiety NRS and 6-month KSS	QUIPS	Q1: M; Q2: M; Q3: M; Q4: L; Q5: M; Q6: L	Uncertain validity of situation-specific anxiety items; follow-up-dependent exclusions; residual confounding despite baseline KSS adjustment.	The validity of context-specific discharge-anxiety items was incompletely established (Q3 Moderate). Follow-up-dependent exclusions and remaining clinical confounding affect Q2/Q5 Moderate.
Jiménez Ortiz 2020	Prospective prognostic cohort	Preoperative anxiety / depression groups and 1-year VAS/KSS	QUIPS	Q1: M; Q2: L; Q3: L; Q4: L; Q5: H; Q6: M	Unadjusted clinical comparisons; key baseline clinical and psychological confounders not controlled.	Group comparisons were primarily unadjusted for key baseline clinical and psychological covariates (Q5 High), limiting independent prognostic interpretation.
Terradas-Monllor 2024	Prospective prognostic cohort	POD7 psychological symptoms and chronic postsurgical pain at 6 months	QUIPS	Q1: M; Q2: M; Q3: L; Q4: L; Q5: M; Q6: H	Same-sample variable selection/ROC analyses without independent validation; residual confounding and uncertain missingness.	Within-sample variable selection and ROC analyses were not externally validated (Q6 High). Missingness and residual confounding were uncertain (Q2/Q5 Moderate).

Study	Study design	Selected outcome/result	Appraisal tool	Risk-of-bias judgement	Main methodological concern(s)	Domain-level judgments and supporting rationale
Larach 2023	Prospective cohort, secondary prognostic analysis	Preoperative anxiety/negative affect and opioid use at 6 weeks/6 months	QUIPS	Q1: M; Q2: L; Q3: L; Q4: L; Q5: M; Q6: M	Model-dependent opioid adjustment; sparse long-term opioid events; exploratory prediction without external validation.	Anxiety–opioid associations depended on adjustment for baseline opioid use (Q5 Moderate). Few long-term opioid events and exploratory prediction limit analysis stability (Q6 Moderate).
JBI (n = 10)						
Bakırhan 2023	Analytical cross-sectional study	TSK associations with pain, strength and sit-to-stand performance ≥4 years after TKA	JBI cross-sectional	No: J5, J6	Confounders not prespecified or adjusted; bivariate correlations only.	Potential confounders such as years since surgery, depression, comorbidity and baseline function were not prespecified (J5 No); only bivariate Pearson correlations were performed, without multivariable adjustment (J6 No).
Turhan Damar 2018	Analytical cross-sectional study	Anxiety/pain and fear of falling at first postoperative mobilization	JBI cross-sectional	Unclear: J7	Insufficient validity/reliability evidence for the single fear-of-falling item.	Validity and reliability of the single-item fear-of-falling outcome were not sufficiently documented (J7 Unclear). Validation of HADS does not establish validation of this outcome item.
Li 2023	Analytical cross-sectional study	Knowledge, support, anxiety and self-efficacy: concurrent serial mediation	JBI cross-sectional	No/Unclear: none	No concerns flagged by No/Unclear responses.	All applicable items were judged Yes in the source cross-sectional checklist; no No or Unclear responses were recorded. This does not imply causality for concurrent mediation analyses.
Zhou 2023	Analytical cross-sectional study	Catastrophizing, kinesiophobia, self-efficacy and exercise adherence: concurrent mediation	JBI cross-sectional	No/Unclear: none	No concerns flagged by No/Unclear responses.	All applicable items were judged Yes in the source cross-sectional checklist; no No or Unclear responses were recorded. Mediation variables were measured concurrently.
He 2023	Analytical cross-sectional study	Postoperative TSK and HADS / self-efficacy associations	JBI cross-sectional	No/Unclear: none	No concerns flagged by No/Unclear responses.	No No or Unclear judgments were recorded in the source cross-sectional checklist. The associations remain cross-sectional.

Study	Study design	Selected outcome/result	Appraisal tool	Risk-of-bias judgement	Main methodological concern(s)	Domain-level judgments and supporting rationale
Cai 2018	Analytical cross-sectional study	Early postoperative TSK and clinical / social / self-efficacy correlates	JBI cross-sectional	No/Unclear: none	No concerns flagged by No/Unclear responses.	No No or Unclear judgments were recorded in the source cross-sectional checklist. The associations remain cross-sectional.
Dahmani 2023	Analytical cross-sectional study	CSI symptoms and GAD-7 / PHQ-9 / pain / disability	JBI cross-sectional	No/Unclear: none	No concerns flagged by No/Unclear responses.	No No or Unclear judgments were recorded in the source cross-sectional checklist. The associations remain cross-sectional.
Priol 2023	Descriptive longitudinal cohort	HADS-A/D trajectories preoperatively, at 6 months and long-term	JBI cohort	No: J4, J5, J10; Unclear: J9	No adequate confounder control or incomplete-follow-up correction; long-term follow-up completeness insufficiently established.	Time-varying postoperative treatments and other confounders were not adequately identified or adjusted (J4/J5 No). Long-term follow-up covered only 65 of 129 participants, with incomplete investigation of missingness (J9 Unclear; J10 No).
Mahdi 2021	Descriptive prospective registry cohort	Preoperative to 1-year HADS-A/D symptom trajectories	JBI cohort	No: J5, J10	Insufficient confounding control and correction for incomplete follow-up.	Baseline clinical confounding of HADS-A/D trajectories was not adequately addressed (J5 No); complete-case analysis lacked a sufficient analytic strategy for incomplete follow-up (J10 No).
İmrek 2025	Uncontrolled single-group pre/post quasi-experiment	Fear VAS before education, after education and before first mobilization	JBI quasi	No: J3, J4, J5, J9; Unclear: J6, J8	Intervening surgery/care; no control group or repeated baseline series; unclear follow-up and fear-VAS reliability; some binary analyses ignore pairing.	Surgery and other care occurred between the education and final fear assessment (J3 No); there was no control group or repeated preintervention series (J4/J5 No). Recruitment/follow-up and fear-VAS reliability were uncertain (J6/J8 Unclear), and some paired data were analyzed as independent counts (J9 No).

Risk-of-bias assessments were conducted using design-appropriate tools. Randomized studies were assessed using RoB 2, non-randomized intervention studies using ROBINS-I, prognostic-factor studies using QUIPS, and observational studies using the relevant JBI critical appraisal checklists. RoB 2 and ROBINS-I judgments are outcome- and comparison-specific. QUIPS results are presented at the domain level, and JBI results are reported according to individual checklist items; therefore, these assessments were not converted into a common overall quality score. Fuchs et al. (2022) was not included in the formal risk-of-bias assessment because the available reporting did not permit sufficiently confident classification of the allocation design. Abbreviations: JBI, Joanna Briggs Institute; QUIPS, Quality In Prognosis Studies; RoB 2, Risk of Bias 2; ROBINS-I, Risk Of Bias In Non-randomized Studies of Interventions. RoB 2 domains: D1, randomization process; D1b, recruitment in cluster-randomized trials; D2, deviations from intended interventions; D3, missing outcome data; D4, measurement of the outcome; D5, selection of the reported result. ROBINS-I domains: I1, confounding; I2, selection of participants; I3, classification of interventions; I4, deviations from intended interventions; I5, missing data; I6, measurement of outcomes; I7, selection of the reported result. QUIPS domains: Q1, study participation; Q2, study attrition; Q3, prognostic factor measurement; Q4, outcome measurement; Q5, study confounding; Q6, statistical analysis and reporting. SC, some concerns; NI, no information. JBI item numbers refer to the specific checklist used.
